# Pillared Vanadium Molybdenum Disulfide Nanosheets: Toward High-Performance Cathodes for Magnesium-Ion Batteries

**DOI:** 10.1021/acsami.3c10287

**Published:** 2023-10-24

**Authors:** Pengcheng Jing, Siobhan Stevenson, Huimin Lu, Peng Ren, Isaac Abrahams, Duncan H. Gregory

**Affiliations:** † WestCHEM, School of Chemistry, Joseph Black Building, University of Glasgow, Glasgow G12 8QQ, U.K.; ‡ School of Material Science and Engineering, Beihang University, Beijing 100083, China; § Department of Chemistry, Queen Mary University of London, Mile End Road, London E1 4NS, U.K.

**Keywords:** magnesium-ion batteries, cathode, vanadium molybdenum sulfide, electrolyte additives, interlayer expansion, nanosheets

## Abstract

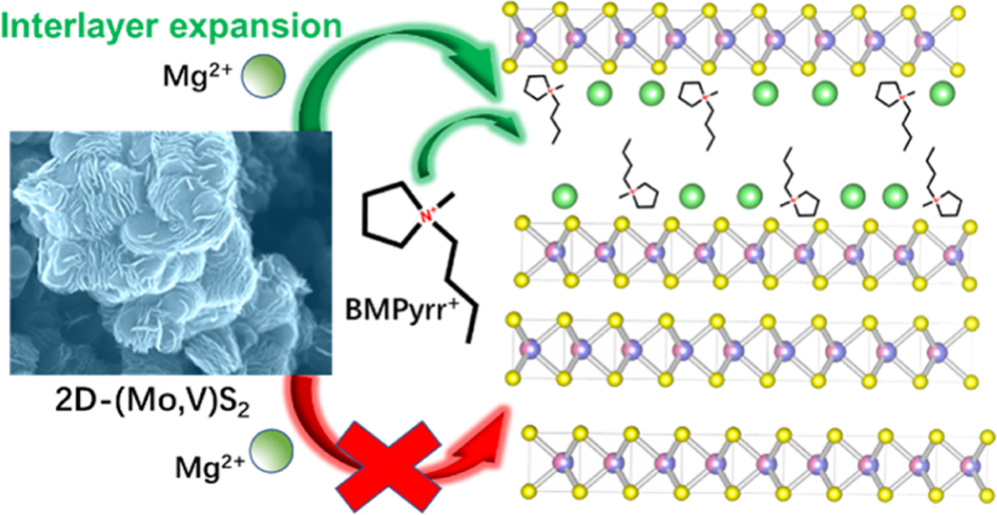

If magnesium-ion batteries (MIBs) are to be seriously considered for next-generation energy storage, then a number of major obstacles need to be overcome. The lack of reversible cathode materials with sufficient capacity and cycle life is one of these challenges. Here, we report a new MIB cathode constructed of vertically stacked vanadium molybdenum sulfide (VMS) nanosheets toward addressing this challenge. The integration of vanadium within molybdenum sulfide nanostructures acts so as to improve the total conductivity, enhancing charge transfer, and to produce abundant lattice defects, improving both the accommodation and transport of Mg^2+^. Additionally, electrolyte additive-induced interlayer expansion provides a means to admit Mg^2+^ cations into the electrode structure and thus enhance their diffusion. The VMS nanosheets are capable of exhibiting capacities of 211.3 and 128.2 mA h g^–1^ at current densities of 100 and 1000 mA g^–1^, respectively. The VMS nanosheets also demonstrate long-term cycling stability, retaining 82.7% of the maximum capacity after 500 cycles at a current density of 1000 mA h g^–1^. These results suggest that VMS nanosheets could be promising candidates for high-performance cathodes in MIBs.

## Introduction

Lithium-ion batteries (LIBs) offer ubiquitous high-energy-density storage options for portable devices and, increasingly, electric vehicles.^1−3^ Use of a Li metal anode provides a means to maximize energy density (with a theoretical capacity of 3860 mA h g^–1^) at low reduction potential (−3.04 V).^4^ However, Li dendrite growth creates safety issues originating from short circuits and thermal runaway.^5,6^ Commercial LIBs circumvent these concerns by replacing Li metal with alternative anodes such as graphite but at the cost of compromising the capacity (372 mA h g^–1^ for LiC_6_).^7^ Future energy storage could be revolutionized by replacing lithium with magnesium. An Mg metal anode exhibits dendrite-free deposition/dissolution across many electrolytes, while also delivering high volumetric capacity (3833 vs 2046 mA h cm^–3^ for Li).^8^ The reduction potential of the Mg couple is slightly less negative (Mg^2+^/Mg −2.37 V), but, crucially, Mg resources in the Earth’s crust are abundant and widespread (while being inexpensive and non-toxic).^9,10^ With these advantages, MIBs have become very attractive for next-generation large-scale energy storage.

A transition to MIBs is not without difficulties, however. One considerable challenge is the design of high-capacity reversible cathodes. The electrostatic interaction between Mg^2+^ and a host lattice is relatively strong such that Mg^2+^ diffusivity is low and reversible discharge–charge capacity is poor in many inorganic materials.^11,12^ Compared to metal oxides, chalcogenides are more likely to cycle Mg^2+^ at a reasonable rate due to the weaker interaction of the anion sublattice with Mg^2+^. As an example, three-dimensional (3D) Mo_6_S_8_, which represented a breakthrough for MIB cathode materials in 2000, can retain 85% of its initial capacity for >2000 cycles.^13^ Two-dimensional (2D) layered MoS_2_ has also been widely studied as an MIB cathode material since nanostructured MoS_2_ was first demonstrated to store Mg^2+^ reversibly in 2004 (with capacities of *ca*. 20 mA h g^–1^).^14^ Although the initial capacities that could be achieved were low, later studies found that they could be improved somewhat by forming nanocomposites either with graphene (yielding 50 mA h g^–1^) or with an MXene (yielding 165 mA h g^–1^).^15,16^ Both the graphene (as an expanded foam) and the Mxene (as delaminated Ti_3_C_2_T_*x*_ flakes) appear to act as electrically conducting supports, enabling further Mg^2+^ to be intercalated into MoS_2_ and consequently increasing the reversible capacity of the disulfide. Neither the graphene foam nor the Mxene, however, were demonstrated to be significantly electrochemically active themselves, with very modest capacities in the absence of MoS_2_. Arguably, a more attractive strategy, therefore, would be to introduce electroactive components that could contribute appreciable additional capacity themselves.

To increase the capacity for Mg ions in a disulfide itself, the 2D interlayer spaces need to be expanded to accommodate magnesium and to facilitate Mg^2+^ diffusion. “Graphene-overlapped” MoS_2_ has been prepared with a large interlayer distance of 1.16 nm. This composite delivered a capacity of 210 mA h g^–1^ at a current density of 20 mA g^–1^, apparently supporting the premise that wider layer separations enable more Mg^2+^ to be stored.^17^ In a slightly different approach, an organic electrolyte additive 1-butyl-1-methylpyrrolidinium ion (denoted hereafter as BMPyrr^+^) could be utilized to expand the layers of a TiS_2_ electrode *in situ* during discharge. The expanded TiS_2_ (which was proposed to form staged co-intercalates of BMPyrr^+^ and MgCl^+^) exhibited relatively fast MgCl^+^ diffusion and delivered a capacity of 239 mA h g^–1^ at 24 mA g^–1^.^18^ A similar method was then applied to the tetrasulfide VS_4_ on reduced graphene oxide (VS_4_@rGO) also using BMPyrrCl (i.e., BMPyrr^+^ cations) as a co-intercalant.^19^ Early cycles showed an obviously improved capacity of 268.3 mA h g^–1^ at 50 mA g^–1^ (as compared to *ca*. 50 mA h g^–1^ for “unexpanded” VS_4_@rGO without BMPyrr^+^).

In principle, the most beneficial additives to MoS_2_ would be expected to improve the electrical conductivity and increase the capacity of the disulfide itself while further making an extrinsic contribution to the overall capacity. Vanadium disulfide has a similar layered structure to MoS_2_ but is metallic while also being able to accommodate Mg^2+^.^12^ VS_2_, therefore, could satisfy the above requirements either as a substituent or as a second component in a composite. Samad and Shin first predicted that monolayers of VS_2_ on a monolayer substrate of MoS_2_ could act as a high-capacity anode for both Li- and Na-ion batteries via density functional theory (DFT) calculations.^20^ Vanadium-substituted MoS_2_ nanoflowers were subsequently synthesized experimentally. The sodium-ion battery (SIB) anodes exhibited improved conductivity compared to MoS_2_ and maintained a capacity in excess of 450 mA h g^–1^ over 800 cycles at 2 A g^–1^.^21^

Herein, we take the above approach of a bimetallic disulfide (VS_2_ – MoS_2_) system that was previously utilized successfully for Na-ion batteries and adapt it to design a new electrode for MIBs. Crucial to the success of this approach is the combination of the concept with the aforementioned interlayer expansion strategy. Correspondingly, we successfully fabricated vertically stacked vanadium–molybdenum sulfide (VMS) nanosheets to be used as a cathode material for MIBs. The VMS nanosheets thus exhibit better conductivity than bare MoS_2_ while also adopting a larger interlayer distance than either MoS_2_ or VS_2_ through the addition of the organic electrolyte additive 1-butyl-1-methylpyrrolidinium chloride (BMPyrr^+^Cl^–^). The effect of these improvements is to enable more magnesium to be stored (i.e., increasing the gravimetric capacity) while also enhancing cation diffusion and improving reversibility. As a result, the expanded VMS nanosheet cathode shows high reversible capacity and long-term stability across varying charging rates.

## Experimental Section

All of the experiments described below were performed at room temperature unless otherwise noted.

## Synthesis of VMS Nanosheets

The synthesis of VMS nanosheets was performed by adapting the procedure previously described by Yue et al. to prepare anodes for SIBs.^22^ 0.468 g of NH_4_VO_3_ (99.5%, Innochem) and 0.7258 g of Na_2_MoO_4_·*x*H_2_O (99.5%, Sigma-Aldrich) were added to 60 mL of distilled water (DW, produced on Millipore Purification System) to which 0.5 mL of aqueous ammonia solution (28.0–30.0 wt % of NH_3_, Alfa Aesar) was added dropwise. 4.8 g of thioacetamide (TAA; C_2_H_5_NS, 99.0%, Aladdin) was then added to the above solution. The solution was sonicated (160 W, Jielimei) for 30 min prior to stirring at 800 rpm for a further 30 min. The dark brown solution obtained from the above procedure was sealed in a Teflon-lined autoclave (100 mL volume, max. *T* = 260 °C, max. *P* = 30 bar, CHEM^N^) and was heated at 220 °C for 24 h. A black solid product was collected by centrifuging (5000 rpm, 3 min), washed three times each with DW and anhydrous ethanol (99.7%, Innochem), and dried under vacuum (0.1 mbar) in a drying oven at 65 °C overnight. The final product was obtained after heating to 300 °C (5 °C min^–1^) and dwelling for 1 h under flowing Ar (99.999%, Dongfangjulong).

Two separate samples were also synthesized to act as controls. First, MoS_2_ was synthesized by using the same approach as above but without the addition of NH_4_VO_3_ as a source of vanadium. Second, VS_2_ was synthesized according to a slightly different but well-accepted literature method.^23^ This latter route was found to be consistently more successful in producing the required product than procedures adopting similar reagents and parameters to the MoS_2_ synthesis. In a typical VS_2_ synthesis, 2.0 g of polyvinylpyrrolidone (PVP, Sigma-Aldrich) was added to 60 mL of DW that contained 4.0 mL of ammonia aqueous solution, after which 0.468 g of NH_4_VO_3_ and 3.0 g of TAA was added. The mixture was stirred at 800 rpm for 1 h to yield a black solution, which was sealed in a Teflon-lined autoclave (100 mL) and heated at 180 °C for 20 h. A black solid product was collected by centrifuging (5000 rpm, 3 min), washed three times each with DW and anhydrous ethanol, and vacuum-dried (0.1 mbar) in a drying oven at 65 °C overnight. The final product was obtained by heating under flowing Ar to 300 °C (5 °C min^–1^) and dwelling for 2 h before cooling naturally to room temperature (RT).

## Materials Characterization

Powder X-ray diffraction (PXRD) patterns were collected in Bragg–Brentano geometry (flat plate; reflection) between 10 ≤ 2θ/° ≤ 70 at 0.1° s^–1^ using either a Bruker *D8 Advance* or a Rigaku *Miniflex* diffractometer with Cu Kα radiation (λ = 0.154 nm; 40 kV, 40 mA). Raman spectra were recorded at RT using a Renishaw *inVia* confocal Raman microscope using a green diode pumped solid state laser with an excitation wavelength of 532 nm operating at 50 mW. Each Raman sample was prepared and measured in air by pressing the relevant dry product onto the surface of a flat glass slide.

The chemical composition of the synthesized samples was measured using inductively coupled plasma-optical emission spectroscopy (ICP-OES, Agilent ICPOES730). Each sample solution was prepared by dissolving 100 mg of the respective product in concentrated nitric acid. Argon was used as the carrier gas and the plasma flow and auxiliary gas flow were 15 and 1.5 L min^–1^, respectively. The axial mode of the instrument was used to detect signals. The thermal stability of VMS samples before and after BMPyrr^+^ intercalation was measured by simultaneous thermogravimetric-differential thermal analysis (TG-DTA) using a Netzsch STA 409 instrument contained within an Ar-filled MBraun UniLab recirculating glovebox (O_2_ and H_2_O < 0.1 ppm). Accurately weighed samples of 15–30 mg were heated in alumina crucibles under a constant flow of Ar (BOC, ≥99.999%, 60 mL min^–1^) from 30 to 500 °C at a 5 °C min^–1^ heating rate.

The Brunauer–Emmett–Teller (BET) surface area and Barrett–Joyner–Halenda (BJH) pore size distribution were determined from nitrogen adsorption data recorded using a Micromeritics ASAP 2460 Surface Area and Porosity Analyzer at −196 °C. Degassing was performed at 300 °C for 6 h. Constituent species and their oxidation states were probed by X-ray photon spectroscopy (XPS) using a Thermo Scientific ESCALAB 250Xi X-ray Photoelectron Spectrometer Microprobe equipped with an Al Kα X-ray source. An Ar^+^ ion beam energy of 4 keV was used for the etching experiments. All high-resolution XP spectra were analyzed and curve-fitted according to Conny and Powell, typically employing dual Gaussian–Lorentzian functions to obtain precise binding energies.^24^ The morphology and spatially resolved elemental composition of the material were characterized using a combination of scanning electron microscopy (SEM; Merlin VP Compact Microscope with a maximum operating voltage of 15 kV) and transmission electron microscopy (TEM; FEI Tecnai G2 F30 Microscope with a maximum operating voltage of 300 kV) each equipped with energy dispersive X-ray spectroscopy (EDS, performed at 15 kV for SEM and 200 kV for TEM). The SEM sample was prepared by scattering dry product powder onto conductive tape, on which a 10 nm thick layer of Au was coated to enhance imaging. The TEM sample was prepared by first mixing approximately 1 mg of dry product powder in 2 mL of ethanol which was then sonicated for 10 min. One droplet of the dispersed powder was then deposited onto the copper TEM grid, which was allowed to dry for 1 h. The microstructural characterization of discharged and charged samples was performed using a Philips/FEI XL30 ESEM instrument (operation voltage 20 kV) with an EDS detector (Oxford Instruments Analytical, U.K.). The discharged and charged electrodes were taken out of the coin cells and twice soaked for 15 min in 3 mL of fresh tetrahydrofuran (THF) followed by drying under vacuum for 5 h at RT. The dry electrodes were affixed on to conductive tabs in the glovebox and quickly transferred to the SEM antechamber from sealed sample vials.

## Electrochemical Tests

The electrochemical behavior of the VMS nanosheets and the 2 control samples, MoS_2_, and VS_2_, was tested by constructing CR2032 coin cells. Mg metal foil pieces (0.2 mm in thickness, diameter of 16 mm, 99.5%, Huabei Magnesium Processing Plant) were employed as the negative electrodes. A positive electrode slurry was made by blending 0.08 g of active material, 0.01 g of conductive carbon (Ketjen black, 99%, Lion Corporation), and 0.01 g of polyvinyldifluorine (PVDF, 98%, average Mw ∼ 534,000, Sigma-Aldrich) binder in *ca*. 0.6 mL of *N*-methyl pyrrolidone (99.5%, Water ≤50 ppm, Innochem). The resulting slurry was coated on to Ni foam chips (99.8%, diameter of 16 mm, thickness of 1 mm, pore diameter of 0.2–0.6 mm, areal density of 280–420 g m^–2^, Saibo Electrochemistry) and dried under vacuum (0.1 mbar) in a drying oven at 60 °C overnight. 0.4 M “All-phenyl complex” (APC) was used as the electrolyte and was prepared by mixing 0.267 g of AlCl_3_ (99%, ultradry, Alfa Aesar) and 2.0 mL of a solution of phenyl magnesium chloride (PhMgCl; 2.0 M in tetrahydrofuran (THF), Macklin) in 3 mL of THF (99.9%, water ≤30 ppm, Innochem). 2 mL of the additive-added electrolyte was obtained by adding 0.089 g of 1-butyl-1-methylpyrrolidinium chloride (BMPyrrCl, 99%, Aladdin) to 2 mL of 0.4 M APC electrolyte (i.e., 0.4 M APC: 0.25 M BMPyrrCl). Discharge and charge cycling and galvanostatic intermittent titration technique (GITT) experiments were performed using a LAND CT2001A battery test system over a cutoff range of 0.2–2.0 V. For GITT experiments, test batteries were allowed to discharge for 600 s at a current density of 50 mA g^–1^, followed by a relaxation period (no current applied) of 1200 s. The discharge/relaxation steps were continued until a limit of 0.2 V was reached. The GITT curve data and information on how they were used to calculate diffusion coefficients are provided in the Supporting Information. Cyclic voltammetry (CV) and electrochemical impedance spectroscopy (EIS) data were collected by using a PalmSense 4 potentiostat at room temperature. CV experiments were conducted over a range of 0.2–2.0 V at scan rates of 0.2–0.8 mV s^–1^. Scans were taken from the open circuit potential to the low-voltage cutoff and then swept to the high-voltage cutoff. EIS experiments were performed over a frequency range of 100 000 to 0.01 Hz with a potential amplitude of 10 mV.

## Results and Discussion

**Figure 1:**
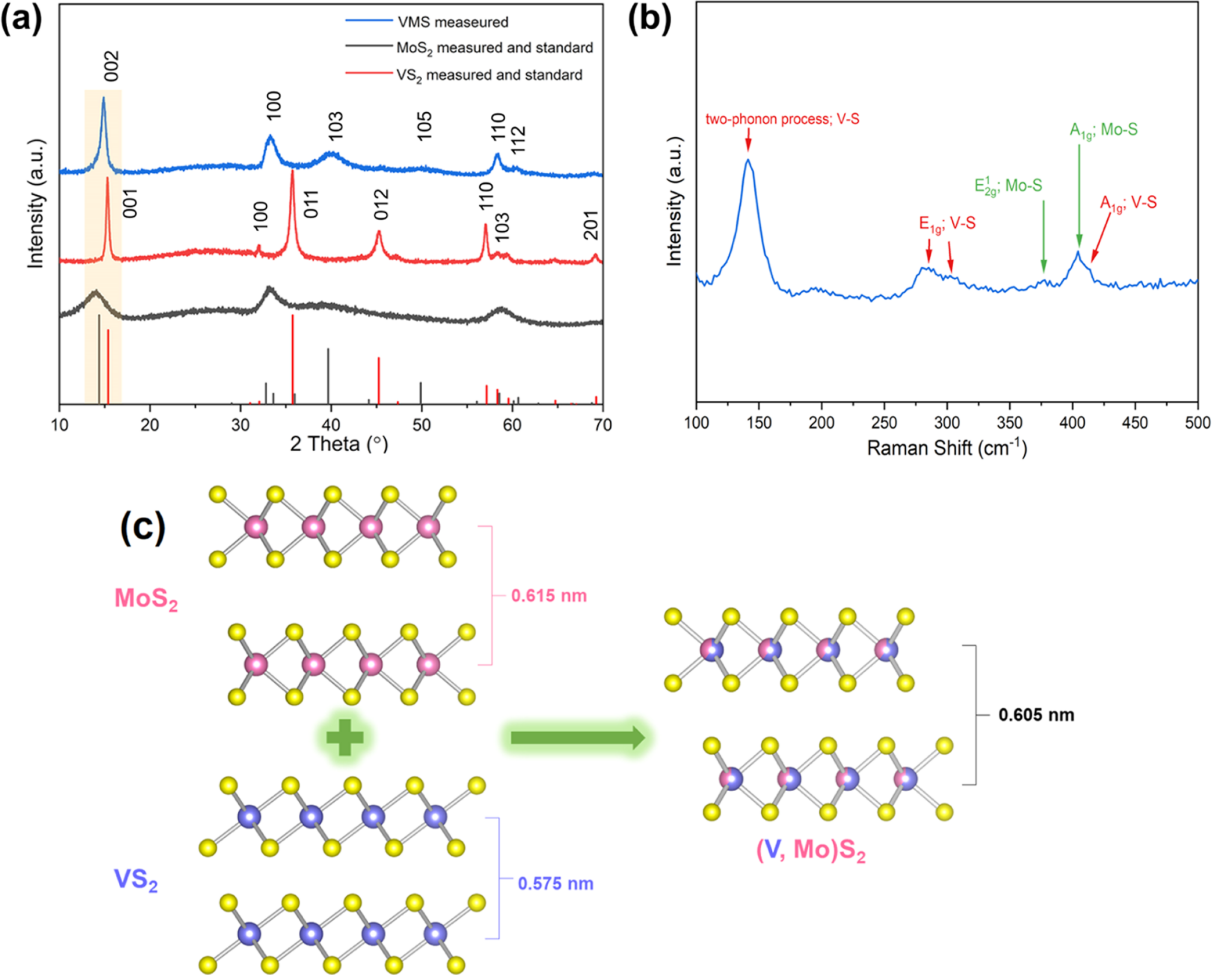
(a) PXRD patterns of the as-prepared VS_2_, MoS_2_, and VMS nanosheet samples. Also shown for reference are PDF entries for 2H-MoS_2_ (PDF-73-1508) and 1T-VS_2_ (PDF-89-1640); (b) Raman spectrum of the VMS nanosheets; (c) schematic representation of the structures of 2H-MoS_2_ and 1T-VS_2_ and the relationship to the 2H-VMS structure.

Figure 1a shows the powder X-ray diffraction (PXRD) patterns of the as-prepared black powders. The strongest intensity peaks in the PXRD pattern of the VMS sample are located at 2θ = 14.8, 33.3, 40.0, and 58.4°, which can be assigned to the (002), (100), (103), and (110) planes of 2H MoS_2_ (PDF-73-1508). The diffraction patterns of the two binary sulfides prepared as control samples match very well to the reflections expected for the 2H structure of MoS_2_ (PDF-73-1508) and the 1T structure of VS_2_ (PDF-89-1640), respectively. From an inspection of the respective diffraction patterns of 2H MoS_2_, 1T VS_2_, and the synthesized VMS material, there are subtle but noticeable shifts in 2θ between the diffractograms of VMS and MoS_2_. The respective peak positions corresponding to the (002) plane of VMS and MoS_2_ (and of the (001) reflection of VS_2_) indicate that the interlayer spacing in VMS is smaller than that of MoS_2_ (but larger than that in VS_2_). Moreover, it should be noted that the VMS diffraction pattern contains no obvious peaks matching to VS_2_ (or any impurities), suggesting that the sample is a single phase. An interlayer distance of 5.95 Å can be obtained from the (002) reflection for VMS (14.85° 2θ), which is intermediate between the values of the equivalent distances of 6.15 Å (14.12° 2θ) and 5.75 Å (15.38° 2θ) for 2H MoS_2_ and 1T VS_2_, respectively. These observations suggest, therefore, that vanadium is substituted into the 2H MoS_2_ structure during hydrothermal synthesis.^21,22^

The Raman spectrum of the as-prepared VMS is shown in Figure 1b. The two bands at approximately 378 and 404 cm^–1^ are due to the in-plane E_2g_^1^ and out-of-plane A_1g_ modes of Mo–S vibrations, respectively.^25^ Both of these bands are also seen in the Raman spectrum of the control sample of 2H MoS_2_ (Figure S1). The two overlapping peaks at *ca*. 281 and 302 cm^–1^ however, can be attributed to the E_1g_ mode as seen in 1T VS_2_, while a peak at *ca*. 406 cm^–1^ resembles the out-of-plane A_1g_ vibration that is also characteristic of VS_2_.^26^ The band at *ca*. 142 cm^–1^ has been previously tentatively identified as being associated with a two-phonon process in VS_2_,^27^ while the broad, low-intensity band at *ca*. 195 cm^–1^ has been observed in spectra of nanostructured VS_2_ on a number of occasions but not previously assigned.^27−29^ Each of these signature V–S bond vibrations is similarly observed in the Raman spectrum of the control sample of 1T VS_2_ (Figure S1). The above results provide further evidence of the presence of both Mo–S and V–S bonds in the VMS nanosheets. Based on the above PXRD and Raman results, Figure 1c gives a schematic representation of the envisaged structural evolution from MoS_2_ and VS_2_ to VMS. From our experimental evidence, VMS is isostructural to MoS_2_ (for example, all of the most intense MoS_2_ reflections are present—and shifted to higher 2θ—while key reflections from the 1T VS_2_ structure are clearly absent in diffraction patterns) which leads to the presumption that V is substituted for Mo within the Mo–S layers. Further evidence of vanadium substitution is discussed in the following sections.

**Figure 2:**
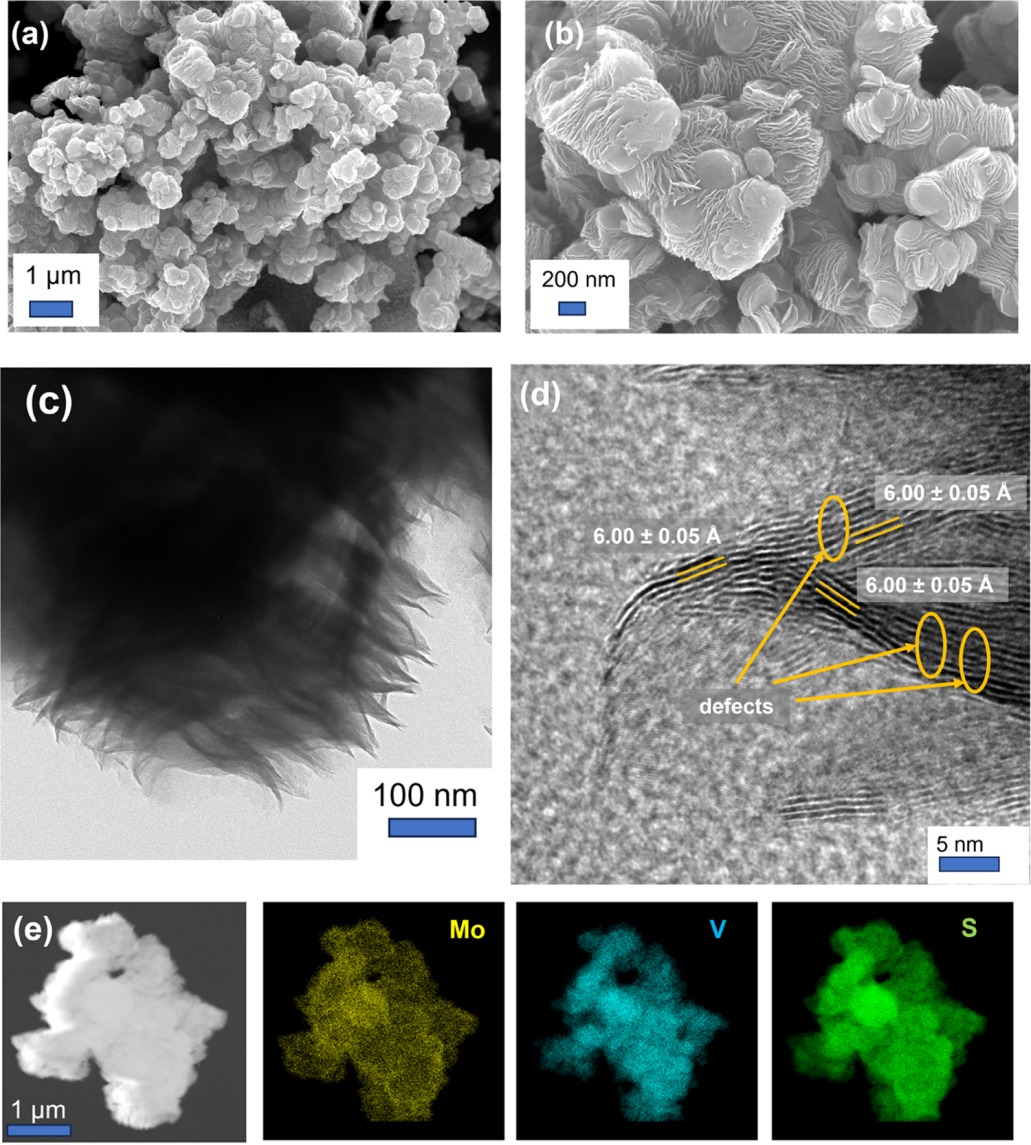
Results of EM experiments on the as-prepared VMS nanosheets showing: (a) low-magnification and (b) high-magnification SEM images; (c) TEM and (d) HRTEM images; and (e) scanning transmission electron microscopy (STEM) image (left) with the corresponding elemental maps for Mo, S, and V, respectively.

SEM and TEM/EDS experiments were performed to characterize the morphology and to verify the composition of the VMS sample. Figure 2 shows the SEM images of the VMS material. The image in Figure 2a demonstrates that the sample can be understood to be a 3D porous assemblage of numerous platelets, which when viewed at higher magnification (Figure 2b) are clearly composed of stacks of many approximately aligned sheets. Each sheet is approximately 100 nm or more across, yet each is considerably thinner in the third dimension. By contrast, the SEM images taken from the control samples of bulk MoS_2_ and VS_2_ (Figure S2) show materials composed of relatively large blocks and of agglomerations of thin nanosheets/flakes (measuring 1–3 μm across), respectively. From the TEM image of the VMS nanosheets portrayed in Figure 2c, it is apparent that each sheet shows some degree of flexibility and that each nanosheet is on the order of 10 nm in thickness. The high-resolution TEM (HRTEM) image in Figure 2d provides a detailed image of the layered structure of a single nanosheet which is apparently composed of approximately ten VMS layers. Measurements of the interlayer spacing (van der Waals gap) taken from different parts of the sample reveal values of 6.00 ± 0.05 Å, which compare to layer spacings of 6.15 and 5.75 Å for 2H MoS_2_ and 1T VS_2_, as obtained from PXRD data taken from the respective control samples. The *d* spacing of 6.00(5) Å is very close to an anticipated value of 5.98 Å obtained by considering the layer separations of the two binary parent materials and from the assumption that a VS_2_–MoS_2_ solid solution obeys Vegard’s law. It is also worth noting that abundant defects, including extended defects such as dislocations, are observed in the HRTEM image, which may play an important role in influencing the charge storage process and mechanism (*e.g*., by providing alternative diffusion pathways and intercalation sites). TEM EDS spectra and corresponding elemental maps for Mo, S, and V are shown in Figures S3 and 2e. The results confirm a V/Mo ratio of 1.3:1 that is consistent with the amounts of the respective starting materials and show that Mo, S, and V are uniformly distributed throughout the sample, which would indicate that V is substituted throughout the MoS_2_ structure. Inductively coupled plasma-optical emission spectroscopy (ICP-OES) was also employed to determine the chemical composition of the VMS sample independently. ICP-OES data (Table S1) imply a stoichiometry of V_0.63_Mo_0.46_S_2_ which equates to a V/Mo ratio of 1.37:1 in entirely satisfactory agreement with the EDS result and consistent with a vanadium-rich, bimetallic sulfide (Mo, V)S_2_.

The Brunauer–Emmett–Teller (BET) surface area and the BJH pore size distribution of the VMS nanosheets and the two disulfide control samples (MoS_2_ and VS_2_) were derived from the nitrogen adsorption data (Figures S4 and S5). The BET specific surface area for the VMS material was calculated to be 13.7 m^2^ g^–1^, while there is a sharp maximum at approximately 2 nm and a broader maximum at 10 nm in the pore diameter distribution of the VMS nanosheets. Beyond this second maximum, there is decreasing but still significant porosity up to diameters of approximately 50 nm. The data therefore suggest both micro- and mesopores are present in the VMS sheets. Considering these data in parallel with the SEM and TEM results, it can be deduced that the values below 20 nm may represent the presence of pores within the nanosheets, whereas the higher values indicate the existence of interparticle porosity.

**Figure 3:**
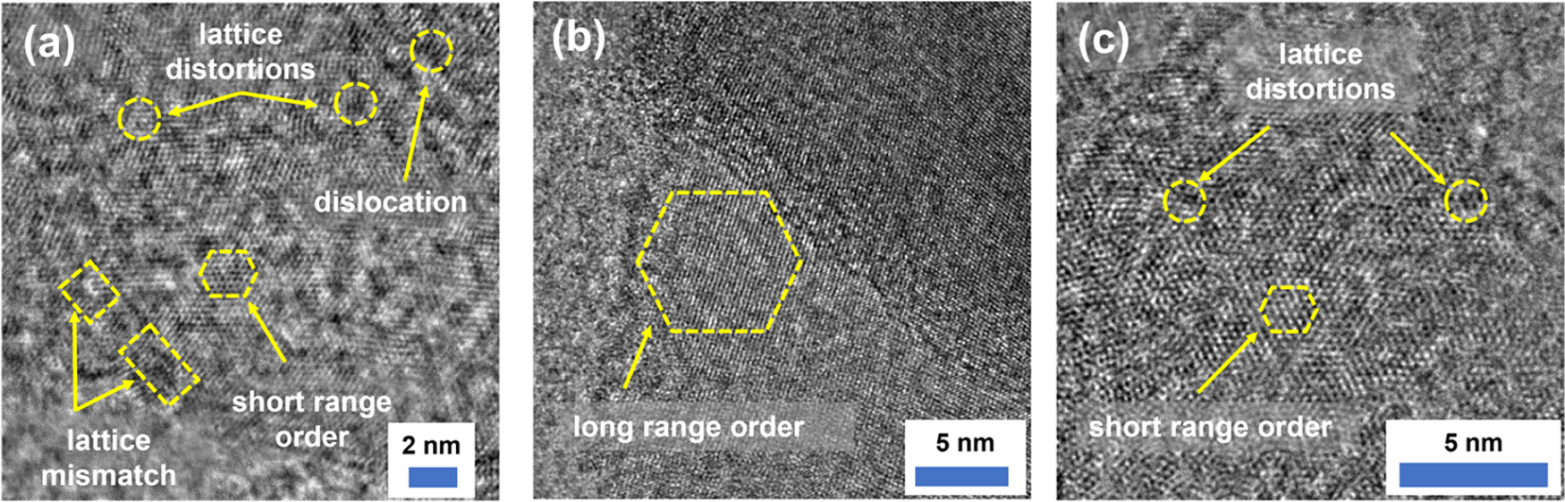
HRTEM images taken from the (a) VMS, (b) VS_2_, and (c) MoS_2_ samples, respectively.

Given that defects may play an important role in providing additional/alternative sites for reaction/inclusion,^30^ HRTEM images of VMS, VS_2_, and MoS_2_ were considered to compare discernible characteristics of their defect structures. From Figure 3, it is apparent that VMS possesses a rich number of extended defects including evidence of dislocations, grain boundaries, and/or lattice mismatch. The highly defective local structure is an unsurprising outcome of the substitution of smaller V for larger Mo in the MoS_2_ framework. In contrast, VS_2_ itself shows long-range order and a smaller number of defects, whereas the sample of MoS_2_ exhibits some evidence of disorder, but is not defective to the same extent as VMS. Hence, the increased concentration of defects that exist following V substitution might not only impinge on the Mg^2+^ diffusion but could also provide additional sites for charge storage.

**Figure 4:**
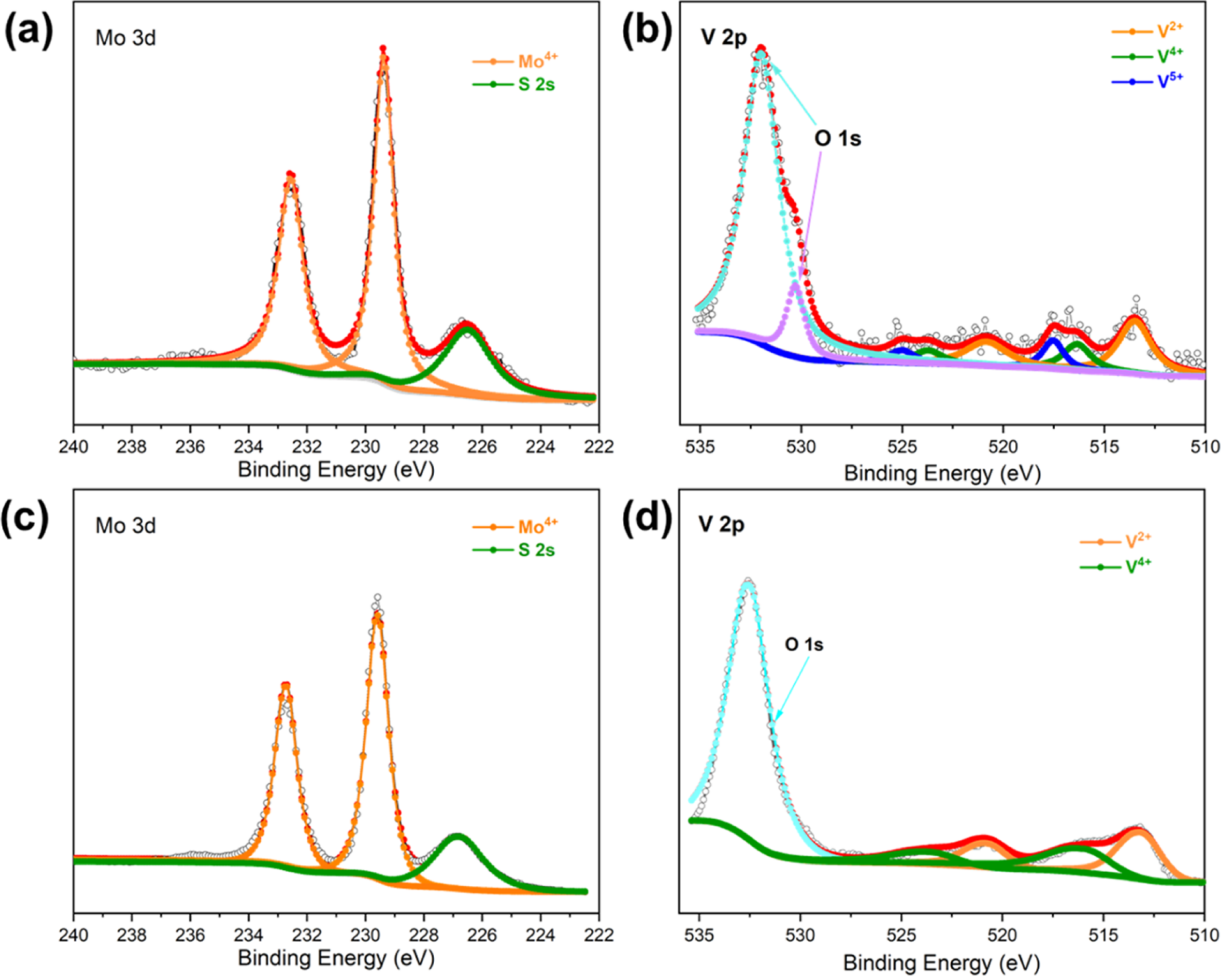
(a) Mo 3d and (b) V 2p regions of the high-resolution XPS spectra taken from VMS nanosheets as compared to (c) Mo 3d and (d) V 2p spectra of MoS_2_ and VS_2_, respectively.

X-ray photon spectroscopy (XPS) was employed to study the surface chemical states of the VMS nanosheets and to make comparisons with the VS_2_, and MoS_2_ control samples. In the Mo 3d high-resolution spectrum for the VMS material (Figure 4a), peaks are located at 232.6 and 229.4 eV, which can be assigned to the Mo 3d_3/2_ and Mo 3d_5/2_ transitions and are placed at binding energies typical for Mo^4+^ in MoS_2_.^16,31,32^ The additional peak at 226.5 eV can be attributed to the S 2s transition, which occurs in the same binding energy region. The value is typical of S^2–^ in MoS_2_.^16^ Moreover, these results are also in good accordance with the Mo 3d spectrum measured for the MoS_2_ control sample shown in Figure 3c. Considering the V 2p spectrum for VMS (Figure 3b), eight peaks are observed in total. The doublet peaks at 523.7 eV of V 2p_1/2_ band and 516.3 eV of V 2p_3/2_ band are ascribed to V^4+^, while the peaks at 520.8 and 513.5 eV can be attributed to V^2+^, which may result from a strong reduction brought about by the thioacetamide present in the reaction mixture.^12,22^ The other V 2p peaks at 525.0 and 517.5 eV are typical for V^5+^. These are proposed to arise from V_2_O_5_ formed from aerial oxidation at the material surface and as corroborated by the presence of O 1s peaks at 532.0 and 530.3 eV.^33,34^ The V 2p spectrum of the VS_2_ sample (Figure 4d) contains very similar signals to that for VMS, with the exception that no convincing evidence for V^5+^ could be found. This may be due to the relatively high stability to oxidation as compared to the highly defective VMS material. The high-resolution S 2p spectrum (Figure S6a) shows 3 pairs of doublet peaks. The first two pairs at 163.6/162.3 eV and at 162.0/161.3 eV can both be assigned to sulfide S^2–^ species. The slight variation in values suggests that the subtly different chemical environments found for metal-sulfide bonds in 2H MoS_2_ and 1T VS_2_, respectively coexist in the VMS materials.^16,31,35−37^ The last pair of doublet peaks at 163.0/162.0 eV can be assigned to S_2_^2–^ species which rationalizes bonding with V^2+^, as was shown to be present in the above V 2p spectra. The S–S bonds created in localized disulfide anions might be expected to facilitate defects that have the potential both to make Mg^2+^ diffusion pathways more favorable and to provide more accessible interstitial cation positions for intercalation. In all other respects, the peaks in the S 2p spectrum of VMS are consistent with those measured for the VS_2_ and MoS_2_ samples (Figure S6b,c).

**Figure 5:**
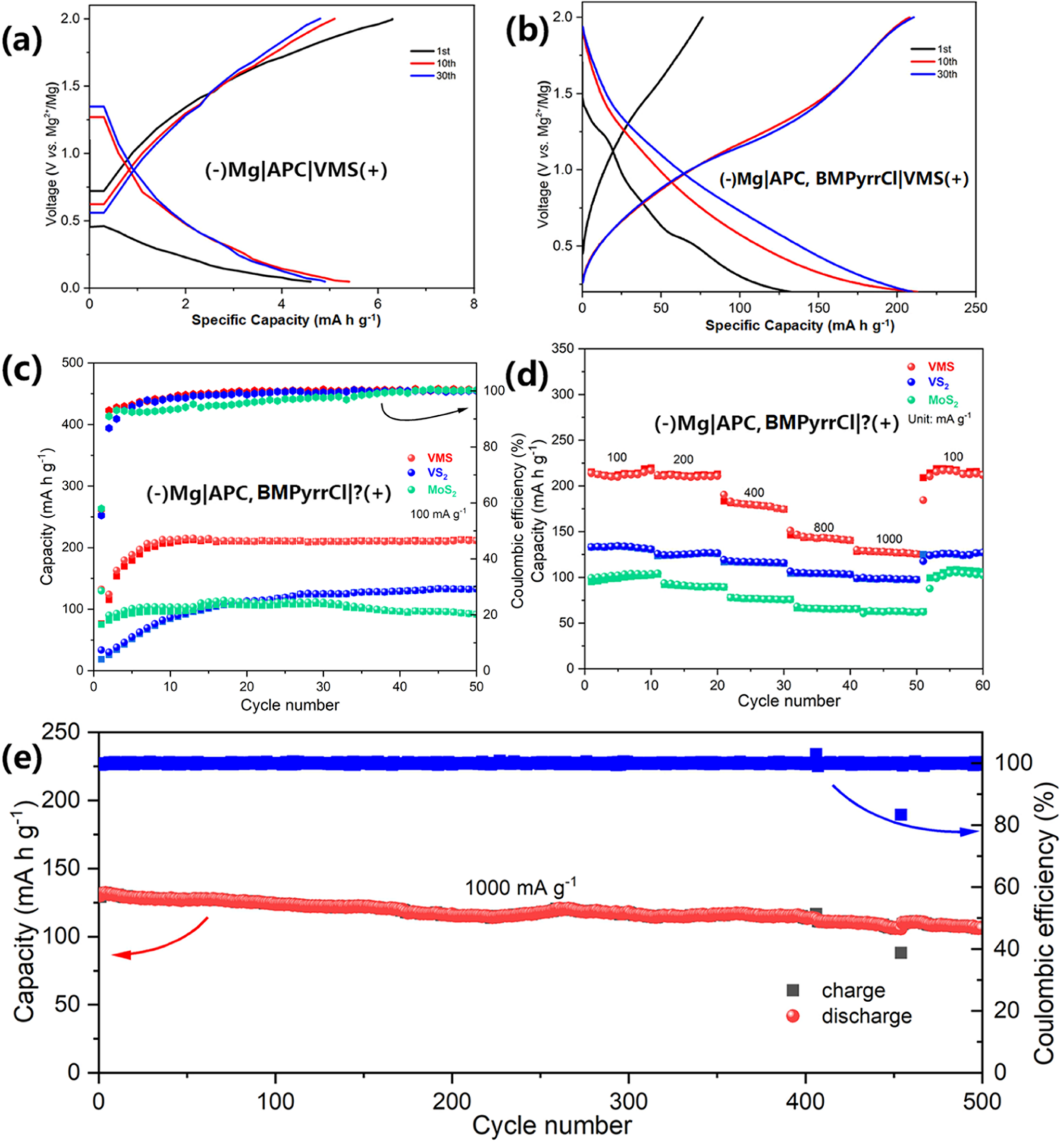
Discharge–charge curves of VMS nanosheets in (a) APC electrolyte and (b) APC-BMPyrrCl electrolyte. (c) Low-current-density cycling measurements of the as-prepared VMS nanosheets vs the VS_2_ and MoS_2_ control samples; (d) subsequent variable (dis)charge rate measurements of the (previously cycled, preactivated) samples shown in (c); (e) subsequent high-current-density cycling measurements of the VMS sample shown in (c) and (d).

The electrochemical performance of the as-prepared VMS nanosheets was determined and compared with the behavior of the control samples of bulk VS_2_ and MoS_2_. A series of experiments was conducted to evaluate: (a) the effect of the vanadium substitution on the MoS_2_ electrode and (b) the significance of the addition of BMPyrrCl to the electrolyte. First, the effect of the electrolyte additive BMPyrr^+^ ions on the performance of the VMS nanosheets was investigated. Importantly, without the addition of the BMPyrr^+^ ions, the VMS nanosheet electrode showed a negligible capacity even if the cell was allowed to discharge to a deep cutoff voltage of 0.05 V (Figure 5a). By comparison, in the presence of BMPyrr^+^ ions, the first discharge capacity increased to 132.4 mA h g^–1^ (Figure 5b). From the profiles of the first discharge curves of the VMS nanosheet electrodes with and without BMPyrrCl added, it would appear that the BMPyrrCl acts so as to activate the nanosheets and improve their ability to store magnesium ions (for example, as Mg^2+^ or as MgCl^+^) in subsequent cycles. It might, therefore, be assumed that the BMPyrr^+^ cations (co)intercalate into and expand the disulfide interlayers, as has been seen in other transition metal disulfides previously; for example, TiS_2_ electrodes.^18^

Cyclic voltammetry (CV) curves were also measured at a scan rate of 0.2 mV s^–1^ (Figure S7). It can be observed that no obvious electrochemical redox peaks are presented in the CV curves of VMS when performed using an *unmodified* APC electrolyte. However, with the addition of BMPyrrCl, three reductive peaks at *ca*. 1.3, 0.9 V, and at the final cutoff voltage, respectively, became apparent. This observation indicates the role of BMPyrr^+^ cations in “activating” the material by way of intercalation-derived interlayer expansion. Upon anodic scanning, one peak at *ca*. 1.4 V is presented, indicating the extraction of Mg^2+^ cations. In the second cycle, one broad reduction peak (*ca*. 1.25 V) which may consist of several reduction peaks and one oxidation peak, similar to that in the first cycle, are observed. Taken completely, the first cathodic scan reveals an irreversible reduction reaction, which may be due to the trapping of BMPyrr^+^ and a limited quantity of Mg^2+^ cations, as can be deduced from the first (dis)charge curves. In the following five scans, the curves ostensibly resemble that obtained from the second cycle yet show gradually strengthened reduction and oxidation currents and lower overpotentials. These features suggest that the electrochemical activation of the materials occurs via accommodation of an increasing amount of Mg^2+^ cations, which is in good accordance with the form of the (dis)charge cycles.

In a second subset of experiments, the effect of varying the transition metal in the disulfide electrode material was examined; in each case using the “optimized” BMPyrr^+^-added electrolyte system. Figure 5c presents the performance of the VMS nanosheet electrode as compared to that of electrodes prepared with bulk VS_2_ or MoS_2_. The cycling behavior of all three electrode materials is relatively stable after an initial activation process in each case. Nevertheless, although each electrode demonstrates levels of stability, the VMS material is distinctive due to its higher reversible capacity of 211.3 mA h g^–1^ compared to 133.1 mA h g^–1^ for VS_2_ and 91.7 mA h g^–1^ for MoS_2_, when (dis)charging at a current density of 100 mA g^–1^. The discharge–charge profiles of the three materials are listed in Figure S8. The cycling performance of the binary materials, VS_2_ and MoS_2_ using the *unmodified* APC electrolyte is also provided (Figure S9). VS_2_ itself presents near negligible capacity during the measurement, whereas MoS_2_ delivers a capacity of *ca*. 100 mA h g^–1^. The reasoning for this difference between the two binary chalcogenide electrodes lies possibly with the larger interlayer spacing of the 4d metal disulfide (6.15 vs 5.75 Å for VS_2_, as taken from our PXRD results above), the material’s ultrathin nanosheet morphology and, as a consequence, its larger surface area as compared to VS_2_. By contrast, however, the cyclic stability of the MoS_2_ electrode is far inferior to that of VS_2_ and the Coulombic efficiency (which can exceed 100%) indicates more parasitic reactions due to the strong direct interaction between MoS_2_ and Mg^2+^ cations.^38−41^

The VMS nanosheets also exhibit a superior rate capability (Figure 5d) compared to that of the binary chalcogenide electrodes. The “preactivated” VMS electrode delivers capacities of 144.5 mA h g^–1^ and 128.2 mA h g^–1^ at increased current densities of 800 and 1000 mA g^–1^, respectively. Figure S10 emphasizes the difference in rate capability for the VMS electrode as compared to the VS_2_ and MoS_2_ bulk electrodes by plotting the respective discharge–charge curves at selected rates from 100 to 1000 mA g^–1^. When considering its long-term cycling performance, the VMS nanosheet electrode exhibits a capacity of 107.5 mA h g^–1^ after 500 cycles at 1000 mA g^–1^. This value corresponds to 82.7% of the activated capacity. The increased capacity and rate performance of the VMS nanosheets over the MoS_2_ and VS_2_ samples likely arises from a combination of heightened structural disorder and hole doping as a consequence of vanadium substitution into the semiconducting 2H-MoS_2_ structure. The possible ramifications would be improved ionic and electrical conductivity, coupled with a higher concentration of available sites for Mg^2+^ intercalation.

**Figure 6:**
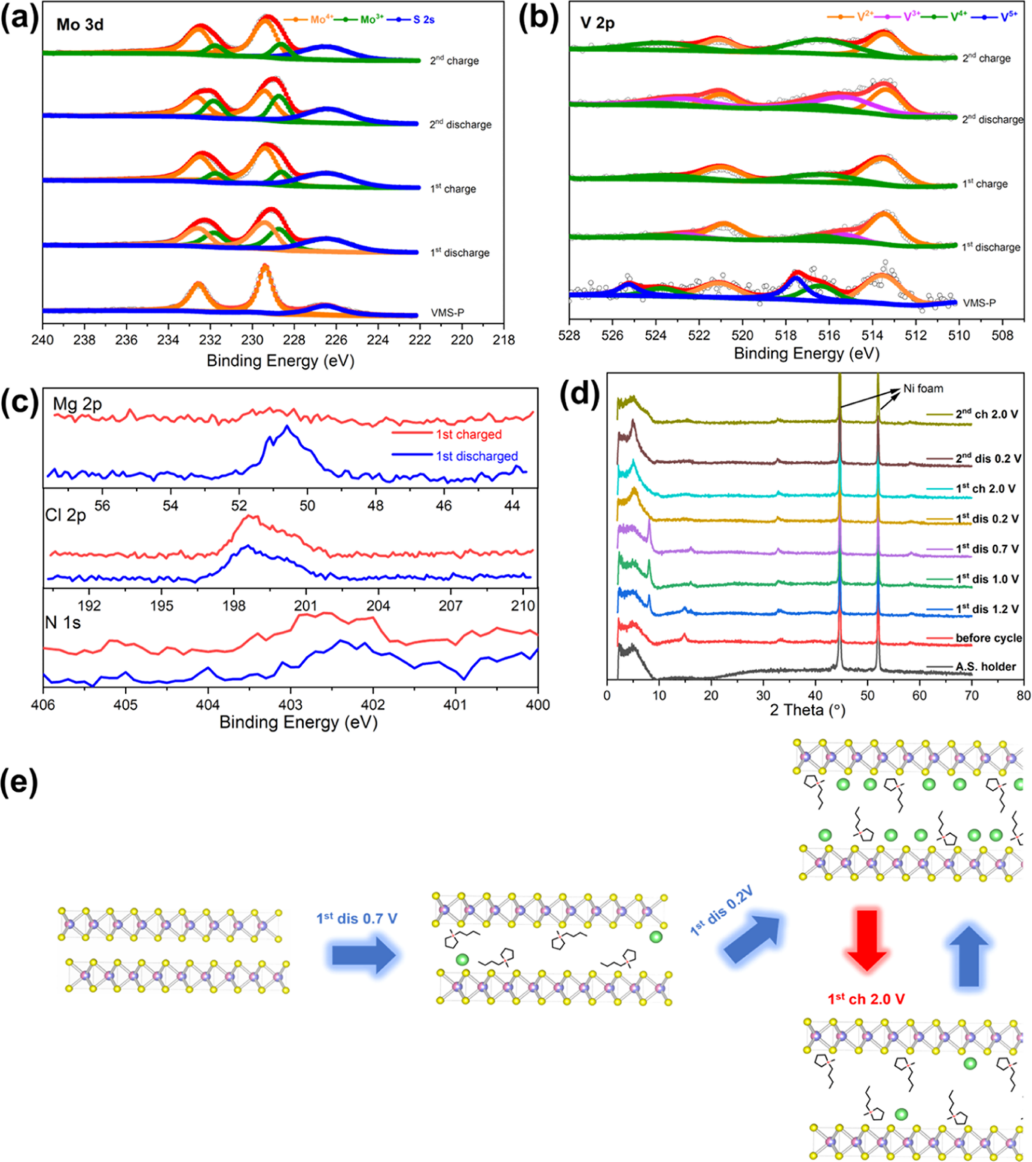
High-resolution XPS spectra for VMS in the (a) Mo 3d, (b) V 2p, and (c) Mg 2p, Cl 2p, and N 1s binding energy regions in the initial charge/discharge cycles. (d) PXRD patterns of VMS nanosheets at different (dis)charge states (the peaks from the Ni current collector are indicated). (e) Schematic illustration of VMS structural evolution during the initial (dis)charge cycles.

In order to probe the compositional and chemical state changes in the VMS electrode material and to establish the likely presence of BMPyrr^+^ ions, a combination of XPS spectra, EDS spectra, and PXRD patterns was collected from the VMS nanosheets in the first two discharge and charge cycles (Figure 6). From the high-resolution XPS Mo 3d spectra of VMS samples, it is clear that the Mo peaks shift to lower binding energies characteristic of Mo^3+^ (231.8/228.6 eV) in the discharged state and partially recover to higher binding energies after full charging (Figure 6a). This indicates a partially reversible Mo^4+^-to- Mo^3+^ redox process during the (de)intercalation of Mg^2+^.^42^ Meanwhile, the V 2p XPS spectra show a similar variation in which V^4+^ is reduced to V^3+^ (522.8/515.4 eV) in the discharged state and reoxidizes to V^4+^ when charged (Figure 6b). Notably, the peak shifts and the corresponding valence state variation become increasingly obvious with cycling, which indicates a steady improvement in reversibility, as is seen in the cycling tests in Figure 5. The N 1s region of the XPS spectrum (Figure 6c) contains a broad, weak intensity peak centered at *ca*. 402.5 eV corresponding to organic N and typical of a pyrroldinium cation.^43^ The signal is thus consistent with BMPyrr^+^ and the peak not only appears in the spectrum of the discharged sample but also remains after fully charging. To probe the presence of BMPyrr^+^ further within the bulk of the electrode material, Ar^+^ beam etching was applied and the corresponding N 1s XPS spectra were measured as a function of etching time/depth in the discharged and charged VMS samples (Figure S11). It was observed that the nitrogen content did not vary significantly over a depth of *ca*. 60 nm in both discharged and charged samples, implying that BMPyrr^+^ is intercalated into the VMS bulk structure during discharge and remains as a pillar in the following cycles. The Mg 2p and Cl 2p regions of the XPS spectra prove informative regarding the nature of the intercalation species. It is notable that the Mg 2p peak reduces in intensity significantly after charging, whereas the Cl 2p signal maintains a similar intensity. The Mg/Cl molar ratios obtained from analysis of the XPS spectra for the discharged and charged VMS nanosheets are approximately 1.6:1 and 0.8:1, respectively, neither of which correspond to the stoichiometries of the known complex Mg–Cl ionic species such as MgCl^+^ and Mg_2_Cl_3_^+^. This mismatch would indicate the possibility of electrolyte adsorption on the surface of the materials despite the thorough washing of the samples with THF. Crucially, EDS results (Tables S2 and S3) show that similar amounts of Al are present in both the discharged and charged samples, which indeed implies electrolyte adsorption, a result that is consistent with observations in the literature.^44^ Given these analytical observations, the principal intercalation species can thus be inferred to be Mg^2+^ rather than MgCl^+^ (and/or Mg_2_Cl_3_^+^).

From a comparison of the PXRD patterns taken from samples at points during the first discharge and charge cycle, respectively (Figure 6d), most remarkable is that the peak present in the pristine material at 14.8° 2θ gradually disappears upon discharging to 1.0 V, while two “new” peaks arise at 8.0° and 15.9° 2θ. The peak positions translate to equivalent *d*-spacings of *ca*. 11.10 and 5.55 Å, respectively. It is tempting to assume from the values of the *d*-spacings and the positions of the peaks in the diffraction patterns that the “new” peaks could be assigned as (00*l*) and (00*2l*) reflections, respectively. On continuing discharge to 0.2 V, it is observed that the peaks at 8.0 and 15.9° 2θ disappear and in the meantime, a new peak arises at approximately 5.2° 2θ (note that this peak has a high intensity above the background of the air-sensitive sample holder), indicating the further expansion of the structure. The features of the diffraction pattern from the sample discharged to 0.2 V suggest progressive Mg^2+^ cation intercalation with the possible rearrangement of VMS layers and their stacking sequences but with the BMPyrr^+^ pillaring species remaining integrated within the structure. While the quality of the diffraction patterns does not permit indexing of the peaks with confidence, it is reasonable to assume that the layered VMS material has expanded significantly following discharge and that this expansion is likely to occur along the crystallographic *c*-direction. TG-DTA was used to test these assumptions by recording the thermal behavior of samples before and after the first discharge (Figure S12). Both samples exhibited some mass loss due to inevitable amounts of residual electrolyte on the surface of the samples; however, the obviously much more substantial mass loss of 18.5 wt % for the first discharged sample indicates the release of BMPyrr^+^ related species from the bulk of the disulfide over a wide temperature range (≤*ca*. 400 °C). The thermal profile of this temperature-dependent release of the organic component is very similar to that observed for layered TiS_2_ co-intercalated with the organic pillaring cation, 1-butyl-1-methylpyrrolidinium, with a relative mass loss commensurate with the heavier MoS_2_ host structure.^18^ The experimental data are thus consistent with the premise of (co)intercalation of BMPyrr^+^ into the van der Waals gap between the transition metal-sulfide layers. The degree of layer expansion and thermal behavior are in keeping with the inclusion of a large, pillaring organic cation, rather than the exclusive incorporation of Mg^2+^ (or Mg–Cl ionic species). Assuming therefore that the mass loss of 13.7 wt % could be attributed to the removal of the intercalated BMPyrr^+^ cations, then the formula of the discharged sample could be approximated as Mg_0.28_(V_0.63_Mo_0.46_)S_2_·0.16 BMPyrr.

The PXRD patterns of the cycle 1 charged sample and both the cycle 2 discharged and charged samples remain ostensibly very similar to that of the material discharged in the first cycle. By the time of the second charge cycle, however, the PXRD patterns display diffraction peaks that are both broader and apparently weaker in intensity suggestive of increased disorder and structures that are progressively transitioning from crystalline to amorphous. The diffractogram of the cycle 2 charged sample appears to show broad peaks at both *ca*. 5.2° 2θ and 7.6° 2θ suggestive of a contraction that could be caused by further extraction of Mg^2+^ ions that were trapped between layers during structural rearrangements in the first cycle. The data would indicate that subsequently, the layered VMS structure does not change significantly further following the removal of Mg^2+^ cations. Therefore, one can assume that the pillaring BMPyrr^+^ cations remain as an integral component of the VMS structure, and indeed the larger organic cations dictate the arrangement of the VMS nanosheets. The interlayer expansion of the VMS structure afforded by the BMPyrr^+^ cations thus proves to be pivotal in facilitating successful Mg^2+^ ion diffusion and is the determinant of whether appreciable Mg can be stored in the electrode or not. One can assume that the VS_2_ and MoS_2_ control samples behave somewhat similarly by undergoing equivalent structural rearrangements. Such assumptions are consistent with prior observations that reversible capacity cannot be achieved in dichalcogenides without the assistance of BMPyrrCl or similar additives.^18,45^

An interpretation of the structural evolution that occurs in VMS in the opening charge–discharge cycles is summarized graphically in Figure 6e. During the discharge to 0.7 V, BMPyrr^+^ is intercalated into the van der Waals gap of the VMS nanosheets accompanied by a degree of Mg^2+^ ion insertion. At this juncture, the arrangement of BMPyrr^+^ is disordered, VMS interlayer expansion is limited and Mg^2+^ has limited access to the interlayer space. Upon further intercalation of BMPyrr^+^ (as the sample is discharged to 0.2 V), there is continued expansion of the (V, Mo)S_2_ layers due to an electrostatically enforced rearrangement of pillaring BMPyrr^+^ cations, which in turn enables further intercalation of Mg^2+^ ions. In subsequent charge/discharge cycles, Mg^2+^ ions can reversibly deintercalate from and intercalate into the VMS scaffold, which remains in an expanded state due to the BMPyrr^+^ pillaring effect.

Following the cycling studies, CV experiments were conducted in an attempt to further understand the charge storage mechanism adopted by the VMS nanosheets. CV curves measured at scan rates of 0.2, 0.4, 0.6, and 0.8 mV s^–1^ are presented in Figure 6a. Equations 1 and 2 were then employed to analyze the experimental data:^46^
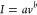
1
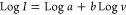
2where *I* is the cathodic/anodic peak current (mA), *v* is the scan rate (mV s^–1^), and *a* and *b* are adjustable constants. Notably, the *b*  parameter can take values from 0.5 to 1.0; when *b* is 1.0 (and *I = av*), the charge storage mechanism is purely capacitance-controlled, while a purely diffusion-controlled process exists for a *b* value of 0.5 (*I* = *av*^1/2^). The measured values for the cathodic and anodic peak currents were collated and plotted as logarithms according to eq 2 (Figure 6b). The linear fits to the data show that the *b* values for the cathodic and anodic processes are 0.88 ± 0.01, 0.92 ± 0.02, 0.99 ± 0.02, and 0.95 ± 0.02 for the R1–3 and O peaks, respectively, indicating that both capacitance- and diffusion-controlled processes contribute to the charge storage mechanism. The *b* values for VMS as compared to those for the two control materials are compiled in Table S4. Capacitance-mediated behavior represents fast Mg^2+^ adsorption on the nanosheet surface and near surface, which allows VMS nanosheets to cycle at high rates, while diffusion-mediated behavior corresponds to the intercalation of Mg^2+^ beyond the surface between the interlayers in the nanosheets.^47^ The VMS nanosheets, similar to previously reported bulk TiS_2_, accommodate bulk organic cations to widen interlayer gaps so as to facilitate Mg^2+^ (de)intercalation. Bulk TiS_2_, however, apparently operates solely through diffusion behavior (*b* = 0.5) with no appreciable capacitance contribution.^18^ Conversely, CV experiments with MIB electrodes fashioned from nanorod assemblies of the spinel Mg(Mg_0.5_V_1.5_)O_4_ yield similar *b* values to the VMS nanosheets (with cathodic and anodic *b* values of *ca*. 0.91–0.96 and 0.99–1.05, respectively).^48^ This finding highlights the important role of nanostructuring (e.g., as nanosheets or nanoparticles) in the surface and near-surface absorption of charge. These capacitance-driven processes by nanostructured electrodes in rechargeable batteries act to enhance the output energy density at high (dis)charge rates. Indeed, similar behavior is observed in other secondary battery chemistries. One powerful example is provided by *n*-octylamine co-intercalated MoSe_2_ nanosheets in sodium-ion batteries (SIBs), where *b* values exceeding 0.9 indicate the dominance of capacitance in the charge storage mechanism.^49^ Another valuable example is provided by CoSe_2_ nanoparticle/carbon nanosheet/MXene composite electrodes in aluminum ion batteries (AIBs) which exhibit *b* values of 0.74 (cathodic) and 0.85 (anodic) when storing Al^3+^ ions;^50^ values that again indicate a diffusion-mediated component is also involved, although this does not dominate the charge storage process. The specific contribution to the capacity from diffusion and capacitance was calculated according to the following equations:^51^
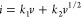
3
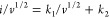
4where *i* is the current (mA) corresponding to a certain voltage (mV) in the CV curve at a specific scan rate *v* (mV s^–1^) and *k*_1_ and *k*_2_ are constants that represent capacitance and diffusion contributions, respectively. The constants *k*_1_ and *k*_2_ were calculated by linear fitting of *i*/*v*^1/2^ against 1/*v*^1/2^, thus allowing the relative proportions of the capacitance and diffusion contributions to be evaluated at different scan rates. The contribution from capacitance-related phenomena in VMS are 82, 85, 88, and 90% at scan rates of 0.2, 0.4, 0.6, and 0.8 mV s^–1^, respectively (Figure 6c). The capacitative contributions are obviously significantly higher than those in VS_2_ and MoS_2_ (Figure S13), indicating fundamentally different charge storage mechanisms in VMS compared to those in the two control materials. Given also the highly disordered crystal structure of VMS and its likely improved electrical conductivity, the combination of surface and bulk defect chemistry in VMS should act so as to facilitate not only high capacity but also fast charge transfer throughout the material. In contrast, VS_2_ which is electrically conducting but relatively defect-free exhibits a lower capacitance contribution than that of VMS. Conversely, MoS_2_, although relatively defective, shows the lowest capacitance contribution of the three materials. It has been previously observed that by forming heterostructures with graphene (with high electronic conductivity), capacitative contributions to charge storage in MoS_2_ become dominant under similar scan rates (92.5%, 1.0 mV s^–1^); both gravimetric capacity and reversibility are also improved over extended cycling.^17^

**Figure 7:**
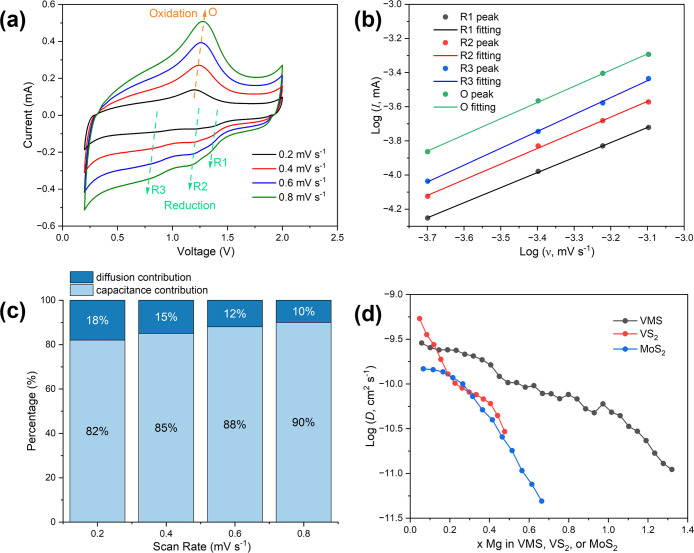
(a) CV curves of the VMS nanosheets taken at different scan rates of 0.2 mV s^–1^ (black), 0.4 mV s^–1^ (red), 0.6 mV s^–1^ (blue), and 0.8 mV s^–1^ (green). (b) Plots of the log cathodic (black, red, and blue) and anodic (green) peak currents against log of the scan rate, *v* for VMS. The solid line represents the linear fit in each case. (c) Plots of the respective capacitance and diffusion contributions to the capacity at different scan rates for VMS. (d) Plots of diffusion coefficients against Mg^2+^ intercalation level for VMS, VS_2_, and MoS_2_ using data derived from GITT curves with cells that had been preactivated.

In order to obtain further understanding of the cationic diffusion in VMS (as compared to VS_2_ and MoS_2_), we conducted a series of galvanostatic intermittent titration technique (GITT) experiments (with details provided in Supporting Information). The diffusion of Mg^2+^ in each of the three materials was investigated enabling the diffusion coefficients (*D*) to be calculated (Figure 7d) based on the data from a series of measured GITT curves (Figure S14). In all three samples, the diffusion coefficients decrease with increasing *x*, commensurate with intercalation behavior and the increasing population of Mg^2+^ in the interlayer gap. It could be observed that the *D* values of VMS range from *ca*. 3 × 10^–10^ to 1 × 10^–11^ cm^2^ s^–1^, which are rather similar to the values measured for Mg^2+^ intercalated into interlayer-expanded TiS_2_.^18^ The diffusion coefficients for VS_2_ and MoS_2_ are somewhat lower than those of the ternary sulfide throughout most of the discharge process. Specifically, for the vast majority of values of intercalation level (*x*), VMS exhibits faster diffusion than both control materials at the same Mg^2+^ cation content, indicating that Mg^2+^ cation transport kinetics is evidently improved in VMS. (The exception is at the smallest, levels, *x* ≤ 0.12, which might be associated with the relative arrangement of the BMPyrr^+^ cations in the interlayer gaps of VS_2_ vs VMS). Altogether, the higher capacitance contribution and better kinetics help boost the cycling capacity and rate performance in the VMS electrodes. Each of these effects is likely to originate from a high concentration of point and extended defects, both at the surface and in the bulk of VMS.

Electrochemical impedance spectroscopy (EIS) measurements were subsequently conducted in order to gain an appreciation of the factors affecting charge transport in the VMS electrode, both before and after apparent activation (achieved after 5 cycles at 100 mA g^–1^). Taking measurements across a frequency limit of 0.01 Hz–100 kHz, Figure S15 and Table S5 show the resulting Nyquist plots and equivalent circuit fits (obtained using AfterMath software) of two representative VMS samples.^52^ A modified Randles circuit model employing an appropriate combination of resistors, capacitors (constant phase elements), and a Warburg impedance (*W*_1_) could be adopted to fit the behavior of the half-cells of both samples. In this model, *R*_1_ represents the bulk (electrolyte and current collector) resistance. The high-frequency semicircle in the Nyquist plots was fitted using a resistance and a capacitive element in parallel (*R*_2_ and constant phase element, CPE_1_) and can be regarded as representing the interfaces (as widely found in metal ion battery systems). The remainder of the profile (fit as *R*_3_ + *W*_1_ and CPE_2_ in parallel) models a mixture of charge transfer and diffusion-controlled behavior in the working electrode as supported by the literature for both Li- and Mg-ion cells.^53,54^ Considering the respective EIS data after fitting, the interfacial resistance decreased sharply from 921 to 12 Ω after the fifth charge. This indicates the modification of the SEI(s) corresponding to “activation” and signals the possible removal of a passivated/oxidized surface layer on the Mg anode and/or on the VMS electrode. With respect to the charge transfer model for the VMS electrode, *R*_3_ dropped significantly to 2933 Ω in the recharged sample from the initial value of 13283 Ω, while the Warburg impedance *W*_1_ in the recharged sample also decreased compared to the initial value (827 vs 285 Ω s^–1/2^). This indicates that charge transfer and Mg^2+^ diffusion in the VSM nanosheet electrode are very slow at the outset but increase dramatically on cycling. The data strongly suggest that following the integration of the organic BMPyrr^+^ cations into the disulfide, the proposed pillaring effect enhances charge transfer to/within the VMS nanosheets and Mg^2+^ diffusion becomes considerably more favorable. Hence, a combination of an expanded interlayer distance in VMS nanosheets (plus a degree of possible layer rearrangement) coupled with the in situ modification of the SEI leads to an increase in capacity over early cycles followed by remarkable stability over an extended duration. Such behavior is seen in several other layered sulfide-based Mg-ion cells where co-intercalated foreign species such as 1-butyl-1-methylpiperidinium in cation intercalated VS_2_,^45^ 2-ethylhexylamine in VS_2_,^12^ and NH_4_^+^ in MoS_2_ act in a similar fashion.^55^

## Conclusions

Vertically stacked vanadium molybdenum sulfide (VMS) nanosheets have been implemented as a cathode material for MIBs for the first time. Vanadium can be incorporated within the 2H structure of MoS_2_ by partially replacing Mo within the metal-chalcogenide layers. The implications of the substitution are essentially 2-fold with evidence of significant disorder introduced into the structure (through point and extended defects) and the likely doping of holes into the valence band of semiconducting MoS_2_. In comparison to samples of the respective binary chalcogenides, VS_2_ and MoS_2_, the VMS nanosheets demonstrate much improved capacity, cyclability, and rate performance when used as cathodes in Mg^2+^ ion cells. The highly defective VMS nanostructures are thus believed to facilitate charge transfer and Mg^2+^ storage coupled with enhanced electrical and ionic transport properties. Nevertheless, crucial to the successful operation of the VMS cathode is the presence of the electrolyte additive BMPyrrCl. Experimental evidence points to a widening of the interlayer spaces in the substituted disulfide brought about by the co-intercalation of BMPyrr^+^. This interlayer expansion provides a route for the Mg^2+^ cation to be (de)intercalated and to diffuse relatively unimpeded within the disulfide structure. As a consequence of vanadium substitution and BMPyrr^+^ interlayer pillaring, the VMS nanosheets exhibit a high reversible capacity and long-term stability. These findings suggest that a combined approach of metal substitution and organic cation co-intercalation should prove profitable in the design of further intercalation electrodes for MIBs.

## Supporting Information Available

Raman spectra, SEM images, EDS spectrum, ICP-OES results, BET surface areas, XPS spectra, CV curves, (dis)charge cycle performance, TGA curves, and GITT curves of VMS, VS_2_, and MoS_2_ (PDF)
