# Supplementary material for: Pillared Vanadium Molybdenum Disulfide Nanosheets: Toward High-Performance Cathodes for Magnesium-Ion Batteries
Source: ACS Appl Mater Interfaces. 2023 Oct 24;15(44):51036–49. doi: 10.1021/acsami.3c10287 (PMC10636724; doi:10.1021/acsami.3c10287)
Supplement: Supplementary file 1 — am3c10287_si_001.pdf [file am3c10287_si_001.pdf]

Supporting Information

Pillared Vanadium Molybdenum Disulfide Nanosheets; Towards High-performance Cathodes for Magnesium-Ion Batteries

Pengcheng Jing,[a] Siobhan Stevenson,[a] Huimin Lu,[b] Peng Ren,[c] Isaac Abrahams,[c] and Duncan H. Gregory[a]*

*E-mail: Duncan.Gregory@glasgow.ac.uk

[a] WestCHEM, School of Chemistry, Joseph Black Building, University of Glasgow, Glasgow, UK, G12 8QQ

[b] School of Material Science and Engineering, Beihang University, Beijing, China, 100083

[c] Department of Chemistry, Queen Mary University of London, Mile End Road, London E1 4NS, UK, E1 4NS

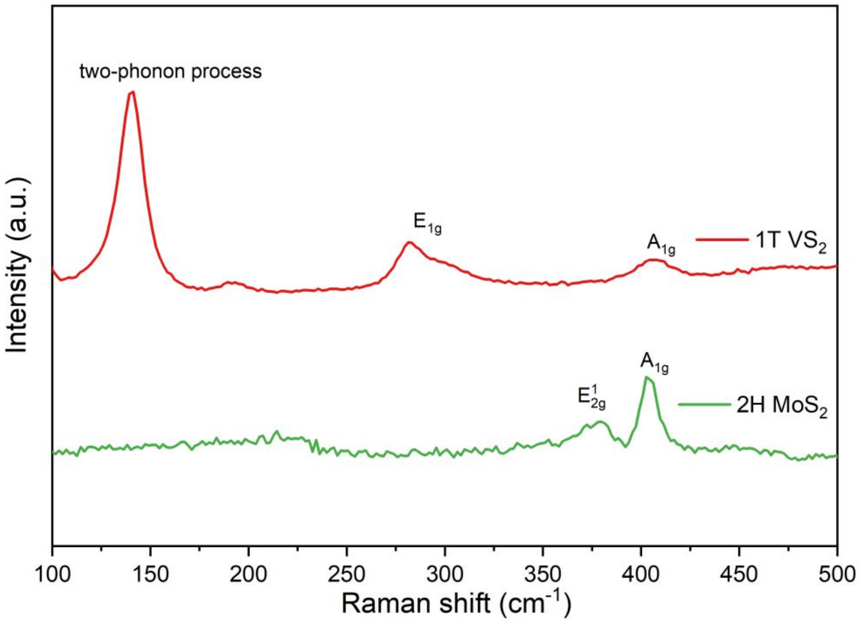

Figure S1. Raman spectra of the as-prepared VS_2 and MoS_2 control samples.

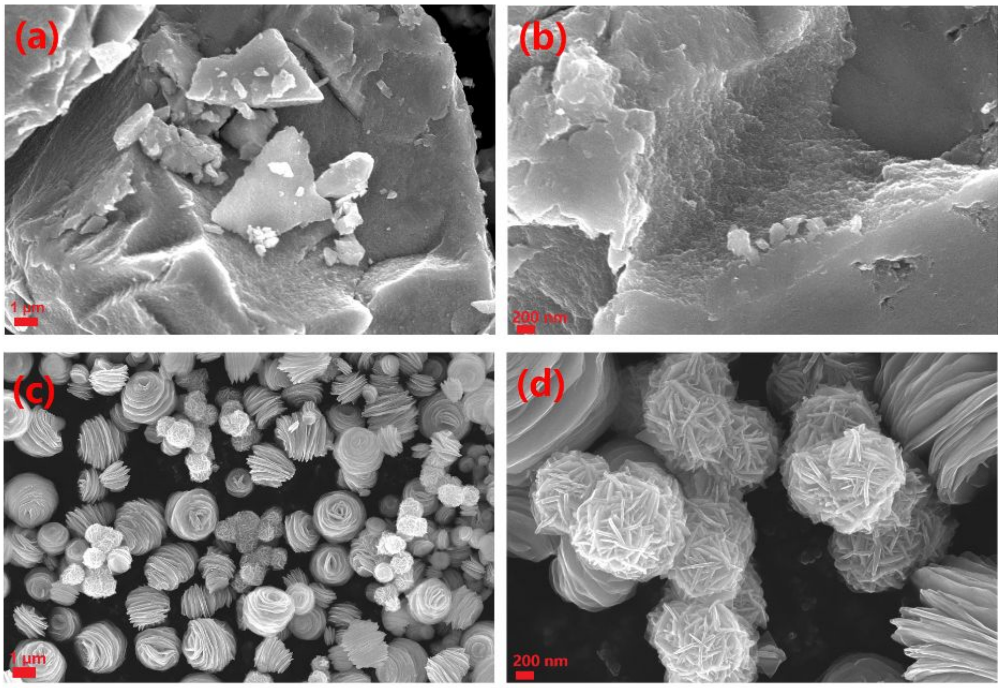

Figure S2. Low and high magnification SEM images of “control” samples of (a)-(b) 2H-MoS_2 and of (c)-(d) 1T-VS_2.

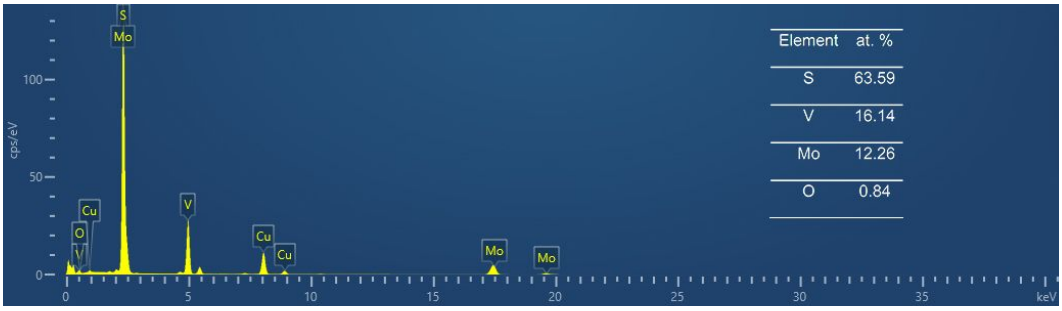

Figure S3. EDS spectrum of VMS nanosheets obtained from TEM measurements. Note that the Cu peaks originate from the Cu sample grid and that the unassigned low intensity signal at *ca*. 5.426 keV can also be attributed to V $K_{\beta1}$.

Table S1. ICP-OES results of the as-prepared VMS nanosheets.

Element	Mass ratio (wt.%)	Atomic ratio (at.%)
Mo	30.89 ± 0.09	14.95 ± 0.19
V	22.34 ± 0.19	20.35 ± 0.12
S	44.58 ± 0.84	64.61 ± 0.22

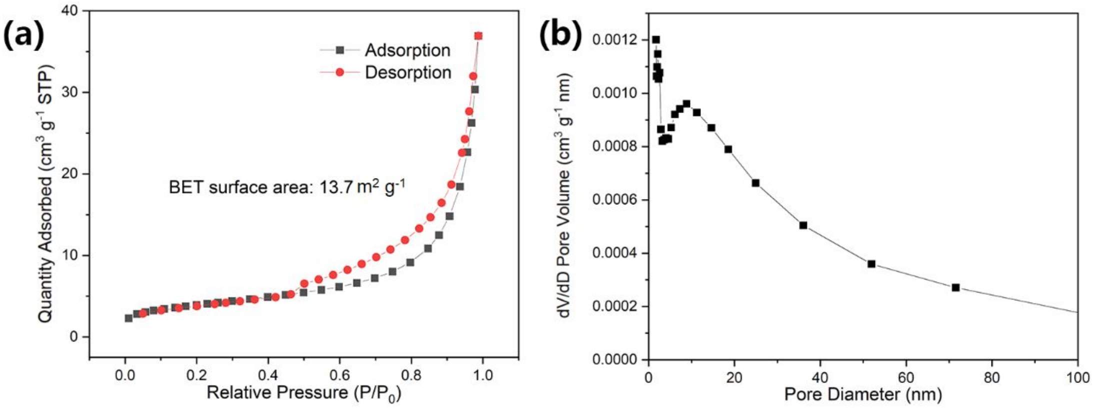

Figure S4. (a) Nitrogen adsorption-desorption isotherm and (b) the BJH pore size distribution curve of the as-prepared VMS nanosheets.

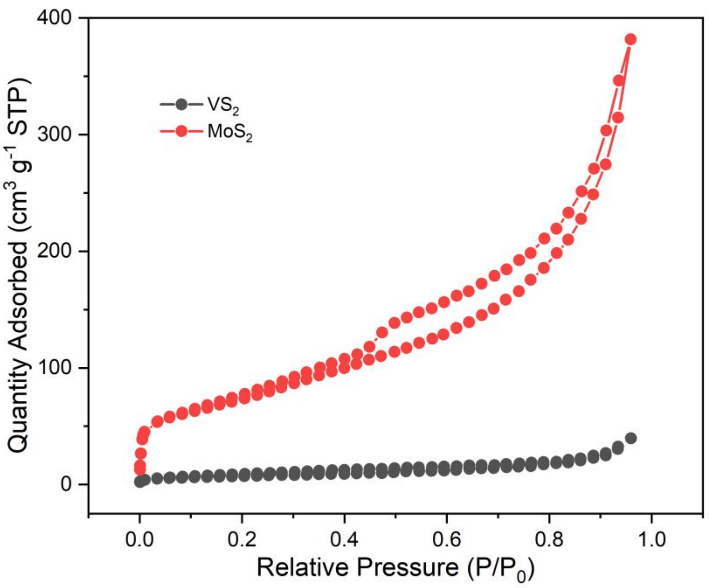

Figure S5. Nitrogen adsorption-desorption isotherm of VS_2 and MoS_2.

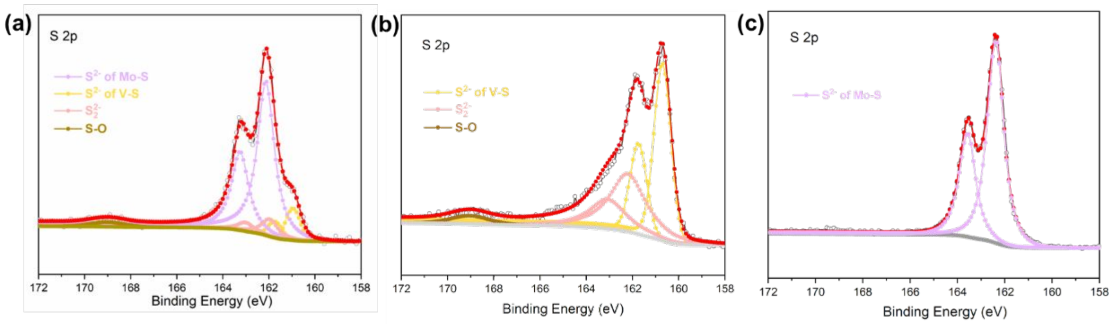

Figure S6. High-resolution S 2p XPS spectra of: (a) the as-prepared VMS nanosheets; (b) the VS_2 control sample and (c) the MoS_2 control sample. Note that the “S-O” label in the figure refers to oxidised SO_4^{2-} species.[1]

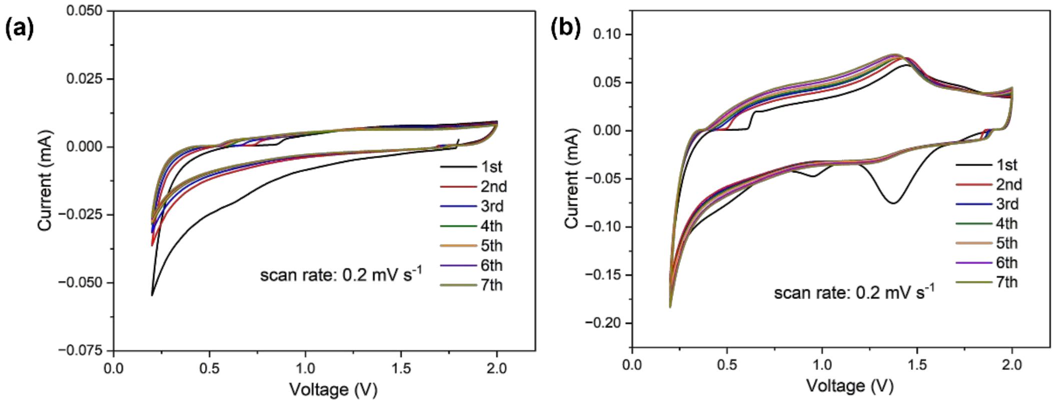

Figure S7. The first seven CV curves of VMS using (a) unmodified APC and (b) APC-BMPyrrCl electrolytes.

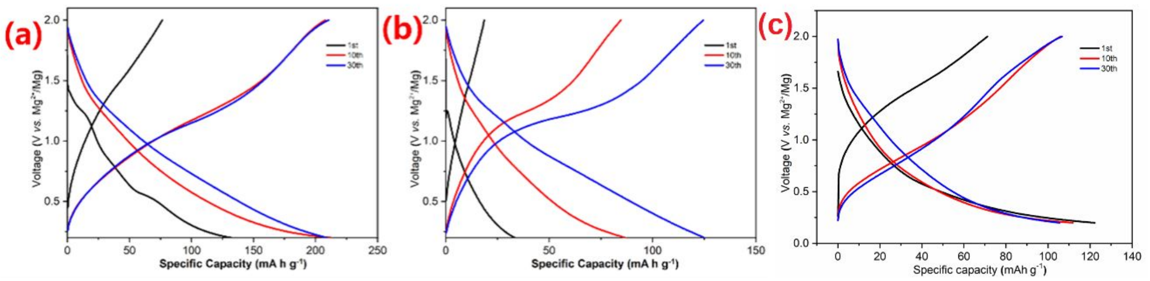

Figure S8. Discharge-charge curves of: (a) the as-prepared VMS nanosheets, (b) VS_2, and (c) MoS_2 at a current density of 100 mA g^{-1}.

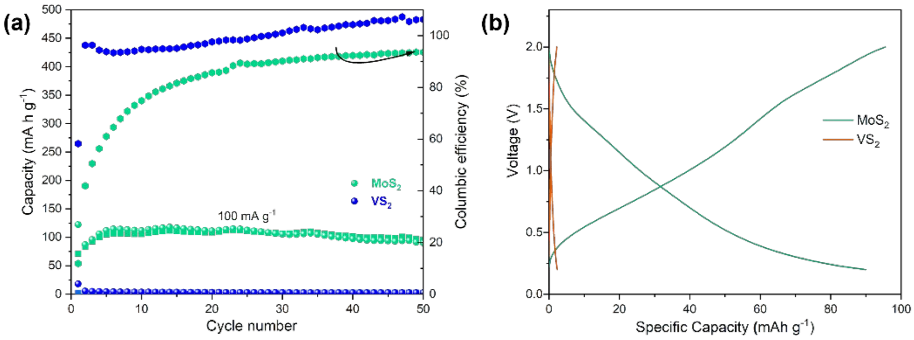

Figure S9. (a) Cyclic performance and (b) 50th (dis)charge curves of VS_2 and MoS_2 using unmodified APC electrolyte at a current density of 100 mA h g^{-1}.

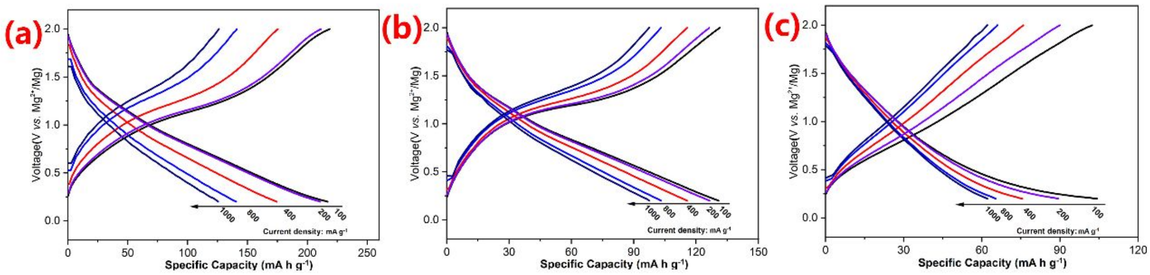

Figure S10. Discharge-charge curves of the precycled, "preactivated" samples of: (a) VMS nanosheets, (b) VS_2, and (c) MoS_2 at current densities of 100 mA g^{-1}, 200 mA g^{-1}, 400 mA g^{-1}, 800 mA g^{-1}, and 1000 mA g^{-1}.

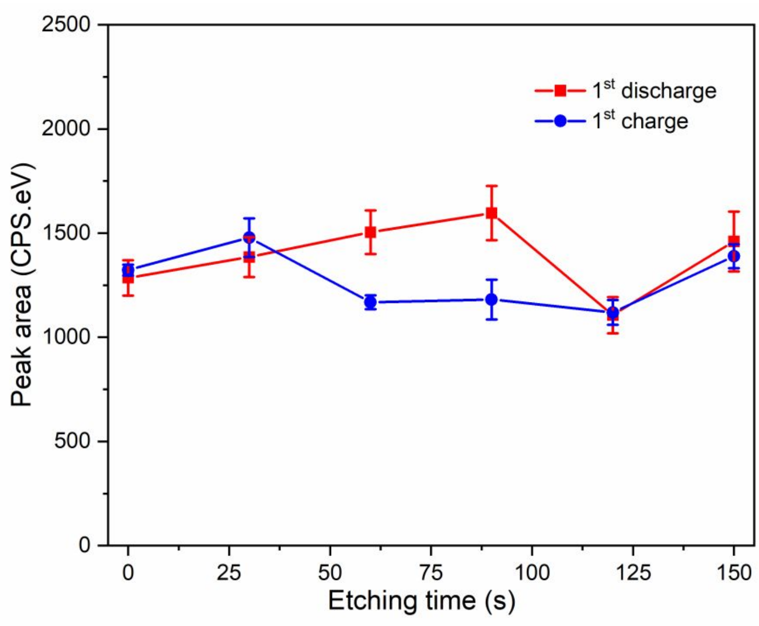

Figure S11. Peak area of N 1s signal (representing the atomic quantity) as a function of etching time (where 150 s represents a depth of *ca*. 60 nm).

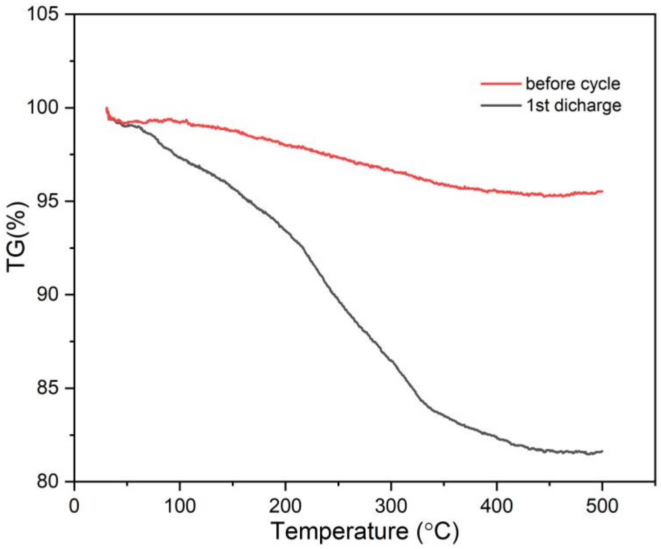

Figure S12. TGA curves of VMS nanosheet samples performed before and after 1st discharge (heating at 5° min^{-1} under flowing Ar gas).

Table S2. EDS results from fully discharged and charged VMS nanosheets.

	As detected by EDS			∴ Originating from residual electrolyte			∴ intercalated (detected – residual)	
Sample	Mg	Cl	Al	Mg	Cl	Al	Mg	Cl
1st discharge	0.585	0.352	0.063	0.126	0.314	0.063	0.460	0.038
1st charge	0.372	0.533	0.096	0.194	0.484	0.096	0.178	0.049

Table S3. Normalised Mg, Cl & Al data[a] (with detected data as taken from Table S2).

	Amount of each element (by emission lines) / at.%							
Sample	C (K)	O (K)	Mg (K)	Al (K)	Cl (K)	V (K)	Mo (L)	S (K)
1st discharge	43.55	17.57	5.5	0.59	3.31	5.37	4.53	18.99
1st charge	57.91	8.66	1.96	0.50	2.81	5.04	4.54	18.24

a. The relative amounts of Mg, Cl, and Al (between 0 and 1) are expressed as the individual at.% values as a fraction of the total (Mg + Cl + Al) content, with all at.% values taken from Table S2. In the 3 columns in Table S3,. "*As detected by EDS*" refers to the normalised amount of each element from analysis of the entire VMS sample area; "*Originating from residual electrolyte*" represents the amount of each element remaining in the electrolyte present outside the bulk of the VMS sample; "*Intercalated*" is equivalent to the amount of Mg and Cl that are intercalated within the bulk of the VMS sample. The *residual* Mg and Cl contents are derived from the value observed for Al assuming that Al cannot be intercalated into the VMS and that from the starting molar ratio of the $PhMgCl{:}AlCl_3$ electrolyte (2:1), the Mg:Al and Cl:Al molar ratios are 2:1 and 5:1, respectively. The *intercalated* Mg and Cl quantities are obtained by subtracting the *residual* values from the *detected* amounts.

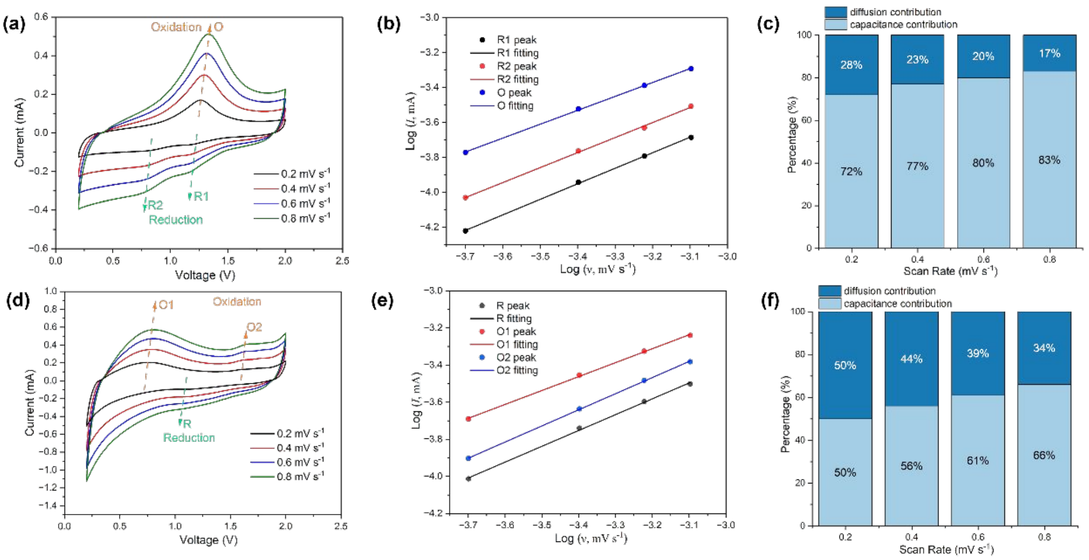

Figure S13. CV curves, plots of the log cathodic (blue) and anodic (red) peak currents against log (*v*, scan rate), and capacitance-diffusion contribution plots of the (a) - (c) VS_2 and (d) - (f) MoS_2 taken at different scan rates of 0.2 mV s^{-1} (black), 0.4 mV s^{-1} (red), 0.6 mV s^{-1} (blue), and 0.8 mV s^{-1} (green), respectively.

Table S4. *b* values of VMS, VS_2, and MoS_2 at reduction and oxidation peaks.

	Peak	*b*-value	Standard Error
VMS	R1	0.88	0.01
	R2	0.92	0.02
	R3	0.99	0.02
	O	0.95	0.02
VS_2	R1	0.89	0.01
	R2	0.86	0.02
	O	0.80	0.01
MoS_2	R	0.85	0.02
	O1	0.75	0.02
	O2	0.87	0.01

Galvanostatic intermittent titration technique (GITT) measurements

The kinetics of Mg^{2+} cation diffusion in VMS, VS_2, MoS_2 were investigated via the GITT method. The diffusion coefficient could be calculated according to the following equation based on Fick's 2nd law: [2]

$$D = \frac{4}{\pi\tau}\left(\frac{m_B V_M}{M_B S}\right)^2\left(\frac{\Delta E_S}{\Delta E_\tau}\right)^2 \quad (1)$$

Where τ, m_B, V_M, and M_B, and S are constant current pulse duration (s), the mass loading (g), the molar volume ($cm^3\ mol^{-1}$), molar mass ($g\ cm^{-3}$), and the contact area of the electrode with the electrolyte (cm^{-2}, normally the coating area of the slurry of the intercalated electrode material on the current collector), respectively. ΔE_τ is the potential change during the current pulse excluding the *IR* drop (where the "IR drop" is the steep potential decrease/increase experienced upon switching on/off the current pulse due to effects including the charge transfer resistances associated with intercalation[3]), and ΔE_s is the potential difference between the equilibrium states before and after the current pulse, both of which could be obtained from the GITT curves (Figure S14).

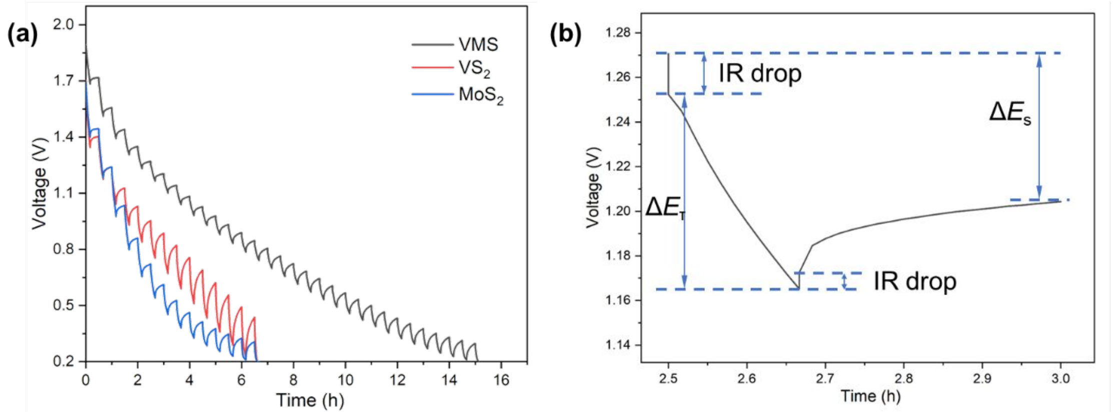

Figure S14. (a) GITT discharge curves of VMS, VS_2, and MoS_2 with 600 s of constant current pulse duration followed by 1200 s of rest time at a current density of 50 mA g^{-1}. (b) One cycle of current pulse and relaxation taken from the GITT curve of VMS shown in (a) indicating the respective ΔE_τ, ΔE_s, and *IR* drop values.

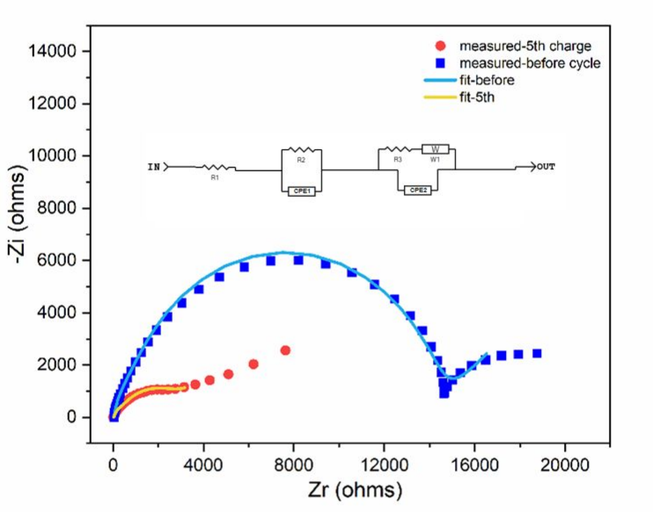

Figure S15. Nyquist plots and respective fits of cells containing electrodes formed from the pristine (blue, light blue) and activated (5 cycles at 100 mA g^{-1}) (red, yellow) VMS nanosheets.

Table S5. Detailed parameters of obtained equivalent circuit model for the two sample cells.

		CPE_1 / S $s^α$	$α_1$ (for CPE_1)	CPE_2 / S $s^α$	$α_2$ (for CPE_2)	R_1/ Ω	R_2 / Ω	R_3 / Ω	W_1 / Ω $s^{-½}$
**before**	value	1.32E-05	0.893684	6.83E-06	0.94854	38.5772	921.093	13283.3	827.133
**cycle**	error	1.39E-07	0.00193295	4.02E-09	0.000131366	0.133385	5.2076	5.42692	0.180534
**5th**	value	4.64E-06	0.979373	0.000400516	0.709575	16.7183	11.7869	2933.73	285.117
**charge**	error	1.23E-06	0.0279006	6.72E-07	0.00076474	0.195204	0.343898	10.0167	3.28224

References:

(1) Guo, T.; Song, Y.; Sun, Z.; Wu, Y.; Xia, Y.; Li, Y.; Sun, J.; Jiang, K.; Dou, S.; Sun, J. Bio-Templated Formation of Defect-Abundant VS_2 as a Bifunctional Material toward High-Performance Hydrogen Evolution Reactions and Lithium−Sulfur Batteries. *J. Energy Chem.* **2020,** *42*, 34-42, DOI: https://doi.org/10.1016/j.jechem.2019.06.007.

(2) Wen, C. J.; Boukamp, B. A.; Huggins, R. A.; Weppner, W. Thermodynamic and Mass Transport Properties of "LiAl". *J. Electrochem. Soc.* **1979,** *126* (12), 2258, DOI: 10.1149/1.2128939.

(3) Knehr, K. W.; Biswas, S.; Steingart, D. A. Quantification of the Voltage Losses in the Minimal Architecture Zinc-Bromine Battery Using Gitt and Eis. *J. Electrochem. Soc.* **2017,** *164* (13), A3101, DOI: 10.1149/2.0821713jes.
